# Supplementary material for: Exenatide once weekly over 2 years as a potential disease-modifying treatment for Parkinson’s disease: protocol for a multicentre, randomised, double blind, parallel group, placebo controlled, phase 3 trial: The ‘Exenatide-PD3’ study
Source: BMJ Open. 2021 May 28;11(5):e047993. doi: 10.1136/bmjopen-2020-047993 (PMC8166598; doi:10.1136/bmjopen-2020-047993)
Supplement: Supplementary data [file bmjopen-2020-047993supp001.pdf]

EUROPEAN COMMISSION
ENTERPRISE AND INDUSTRY DIRECTORATE-GENERAL

Consumer goods
**Pharmaceuticals**

Brussels, 03 February 2010
ENTR/F/2/AM/an D(2010) 3374

**EudraLex**

**The Rules Governing Medicinal Products in the European Union**

**Volume 4**

**EU Guidelines to**

**Good Manufacturing Practice**

**Medicinal Products for Human and Veterinary Use**
**Annex 13**

# Investigational Medicinal Products

| Document History | |
|---|---|
| Revision to reinforce the principle of independence between production and quality control functions in cases where the number of personnel involved is small.<br><br>Changes to sections 36 and 37 to supplement, for investigational medicinal products, the guidance for reference and retention samples given in Annex 19.<br><br>An additional note has been introduced to clarify the meaning of "reconstitution" as referred to in article 9.2 of Directive 2005/28/EC.<br><br>The content of the Batch Certificate referred to in Art. 13(3) of Directive 2001/20/EC, agreed following a separate public consultation, has been added as an attachment.<br><br>A few editorial changes have been made to sections not consulted upon in the interests of updating references and consistency with terminology used throughout the GMP Guide. | February 2008 |
| Public consultation | April 2008 until January 2009 |
| Adopted by the European Commission | 31 January 2010 |
| Deadline for coming into operation | 31 July 2010 |

Commission Européenne, B-1049 Bruxelles / Europese Commissie, B-1049 Brussel – Belgium. Telephone: (32-2) 299 11 11

## PRINCIPLE

Investigational medicinal products should be produced in accordance with the principles and the detailed guidelines of Good Manufacturing Practice for Medicinal Products (The Rules Governing Medicinal Products in The European Community, Volume IV). Other guidelines published by the European Commission should be taken into account where relevant and as appropriate to the stage of development of the product. Procedures need to be flexible to provide for changes as knowledge of the process increases, and appropriate to the stage of development of the product.

In clinical trials there may be added risk to participating subjects compared to patients treated with marketed products. The application of GMP to the manufacture of investigational medicinal products is intended to ensure that trial subjects are not placed at risk, and that the results of clinical trials are unaffected by inadequate safety, quality or efficacy arising from unsatisfactory manufacture. Equally, it is intended to ensure that there is consistency between batches of the same investigational medicinal product used in the same or different clinical trials, and that changes during the development of an investigational medicinal product are adequately documented and justified.

The production of investigational medicinal products involves added complexity in comparison to marketed products by virtue of the lack of fixed routines, variety of clinical trial designs, consequent packaging designs, and the need, often, for randomisation and blinding and increased risk of product cross-contamination and mix up. Furthermore, there may be incomplete knowledge of the potency and toxicity of the product and a lack of full process validation, or, marketed products may be used which have been re-packaged or modified in some way. These challenges require personnel with a thorough understanding of, and training in, the application of GMP to investigational medicinal products. Co-operation is required with trial sponsors who undertake the ultimate responsibility for all aspects of the clinical trial including the quality of investigational medicinal products. The increased complexity in manufacturing operations requires a highly effective quality system.

The Annex also includes guidance on ordering, shipping, and returning clinical supplies, which are at the interface with, and complementary to, guidelines on Good Clinical Practice.

### Notes

*Non-investigational medicinal product*[1]

Products other than the test product, placebo or comparator may be supplied to subjects participating in a trial. Such products may be used as support or escape medication for preventative, diagnostic or therapeutic reasons and/or needed to ensure that adequate medical care is provided for the subject. They may also be used in accordance with the protocol to induce a physiological response. These products do not fall within the definition of investigational medicinal products and may be supplied by the sponsor, or the investigator. The sponsor should ensure that they are in accordance with the notification/request for authorisation to conduct the trial and that they are of appropriate quality for the purposes of the trial taking into account the source of the materials, whether or not they are the subject of

[1] Further information can be found in the European Commission's Guidance on Investigational Medicinal Products (IMPs) and other Medicinal Products used in Clinical Trials

a marketing authorisation and whether they have been repackaged. The advice and involvement of a Qualified Person is recommended in this task.

*Manufacturing authorisation and reconstitution*

Both the total and partial manufacture of investigational medicinal products, as well as the various processes of dividing up, packaging or presentation, is subject to the authorisation referred to in Article 13(1) Directive 2001/20/EC, cf. Article 9(1) Directive 2005/28/EC. This authorisation, however, shall not be required for reconstitution under the conditions set out in Article 9(2) Directive 2005/28/EC. For the purpose of this provision, reconstitution shall be understood as a simple process of:

- dissolving or dispersing the investigational medicinal product for administration of the product to a trial subject,
- or, diluting or mixing the investigational medicinal product(s) with some other substance(s) used as a vehicle for the purposes of administering it,

Reconstitution is not mixing several ingredients, including the active substance, together to produce the investigational medicinal product.

An investigational medicinal product must exist before a process can be defined as reconstitution.

The process of reconstitution has to be undertaken as soon as practicable before administration.

This process has to be defined in the clinical trial application / IMP dossier and clinical trial protocol, or related document, available at the site.

## GLOSSARY

### Blinding

A procedure in which one or more parties to the trial are kept unaware of the treatment assignment(s). Single-blinding usually refers to the subject(s) being unaware, and double-blinding usually refers to the subject(s), investigator(s), monitor, and, in some cases, data analyst(s) being unaware of the treatment assignment(s). In relation to an investigational medicinal product, blinding shall mean the deliberate disguising of the identity of the product in accordance with the instructions of the sponsor. Unblinding shall mean the disclosure of the identity of blinded products.

### Clinical trial

Any investigation in human subjects intended to discover or verify the clinical, pharmacological and/or other pharmacodynamic effects of an investigational product(s) and/or to identify any adverse reactions to an investigational product(s), and/or to study absorption, distribution, metabolism, and excretion of one or more investigational medicinal product(s) with the object of ascertaining its/their safety and/or efficacy.

**Comparator product**

An investigational or marketed product (i.e. active control), or placebo, used as a reference in a clinical trial.

**Investigational medicinal product**

A pharmaceutical form of an active substance or placebo being tested or used as a reference in a clinical trial, including a product with a marketing authorisation when used or assembled (formulated or packaged) in a way different from the authorised form, or when used for an unauthorised indication, or when used to gain further information about the authorised form.

**Investigator**

A person responsible for the conduct of the clinical trial at a trial site. If a trial is conducted by a team of individuals at a trial site, the investigator is the responsible leader of the team and may be called the principal investigator.

**Manufacturer/importer of Investigational Medicinal Products**

Any person engaged in activities for which the authorisation referred to in Article 13(1) of Directive 2001/20/EC is required.

**Order**

Instruction to process, package and/or ship a certain number of units of investigational medicinal product(s).

**Product Specification File**

A reference file containing, or referring to files containing, all the information necessary to draft the detailed written instructions on processing, packaging, quality control testing, batch release and shipping of an investigational medicinal product.

**Randomisation**

The process of assigning trial subjects to treatment or control groups using an element of chance to determine the assignments in order to reduce bias.

**Randomisation Code**

A listing in which the treatment assigned to each subject from the randomisation process is identified.

**Shipping**

The operation of packaging for shipment and sending of ordered medicinal products for clinical trials.

**Sponsor**

An individual, company, institution or organisation which takes responsibility for the initiation, management and/or financing of a clinical trial.

## QUALITY MANAGEMENT

1. The Quality System, designed, set up and verified by the manufacturer or importer, should be described in written procedures available to the sponsor, taking into account the GMP principles and guidelines applicable to investigational medicinal products.

2. The product specifications and manufacturing instructions may be changed during development but full control and traceability of the changes should be maintained.

## PERSONNEL

3. All personnel involved with investigational medicinal products should be appropriately trained in the requirements specific to these types of product.

Even in cases where the number of staff involved is small, there should be, for each batch, separate people responsible for production and quality control.

4. The Qualified Person should ensure that there are systems in place that meet the requirements of GMP and should have a broad knowledge of pharmaceutical development and clinical trial processes. Guidance for the Qualified Person in connection with the certification of investigational medicinal products is given in paragraphs 38 to 41.

## PREMISES AND EQUIPMENT

5. The toxicity, potency and sensitising potential may not be fully understood for investigational medicinal products and this reinforces the need to minimise all risks of cross-contamination. The design of equipment and premises, inspection / test methods and acceptance limits to be used after cleaning should reflect the nature of these risks. Consideration should be given to campaign working where appropriate. Account should be taken of the solubility of the product in decisions about the choice of cleaning solvent.

## DOCUMENTATION

### Specifications and instructions

6. Specifications (for starting materials, primary packaging materials, intermediate, bulk products and finished products), manufacturing formulae and processing and packaging instructions should be as comprehensive as possible given the current state of knowledge. They should be periodically re-assessed during development and updated as necessary. Each new version should take into account the latest data, current technology used, regulatory and pharmacopoeial requirements, and should allow traceability to the previous document. Any changes should be carried out according to a written procedure, which should address any implications for product quality such as stability and bio equivalence.

7. Rationales for changes should be recorded and the consequences of a change on product quality and on any on-going clinical trials should be investigated and documented[2].

**Order**

8. The order should request the processing and/or packaging of a certain number of units and/or their shipping and be given by or on behalf of the sponsor to the manufacturer. It should be in writing (though it may be transmitted by electronic means), and precise enough to avoid any ambiguity. It should be formally authorised and refer to the Product Specification File and the relevant clinical trial protocol as appropriate.

**Product Specification File**

9. The Product Specification File (see glossary) should be continually updated as development of the product proceeds, ensuring appropriate traceability to the previous versions. It should include, or refer to, the following documents:

- Specifications and analytical methods for starting materials, packaging materials;
- Intermediate, bulk and finished product;
- Manufacturing methods;
- In-process testing and methods;
- Approved label copy;
- Relevant clinical trial protocols and randomisation codes, as appropriate;
- Relevant technical agreements with contract givers, as appropriate;
- Stability data;
- Storage and shipment conditions.

The above listing is not intended to be exclusive or exhaustive. The contents will vary depending on the product and stage of development. The information should form the basis for assessment of the suitability for certification and release of a particular batch by the Qualified Person and should therefore be accessible to him/her. Where different manufacturing steps are carried out at different locations under the responsibility of different Qualified Persons, it is acceptable to maintain separate files limited to information of relevance to the activities at the respective locations.

**Manufacturing Formulae and Processing Instructions**

10. For every manufacturing operation or supply there should be clear and adequate written instructions and written records. Where an operation is not repetitive it may not be

[2] Guidance on changes that require the request of a substantial amendment to the IMP dossier submitted to the Competent Authorities is given in the CHMP guideline on the Requirements to the Chemical and Pharmaceutical Quality Documentation Concerning Investigational Medicinal Products in Clinical Trials

necessary to produce Master Formulae and Processing Instructions. Records are particularly important for the preparation of the final version of the documents to be used in routine manufacture once the marketing authorisation is granted.

11. The information in the Product Specification File should be used to produce the detailed written instructions on processing, packaging, quality control testing, storage conditions and shipping**.**

**Packaging Instructions**

12. Investigational medicinal products are normally packed in an individual way for each subject included in the clinical trial. The number of units to be packaged should be specified prior to the start of the packaging operations, including units necessary for carrying out quality control and any retention samples to be kept. Sufficient reconciliations should take place to ensure the correct quantity of each product required has been accounted for at each stage of processing.

**Processing, testing and packaging batch records**

13. Batch records should be kept in sufficient detail for the sequence of operations to be accurately determined. These records should contain any relevant remarks which justify the procedures used and any changes made, enhance knowledge of the product and develop the manufacturing operations.

14. Batch manufacturing records should be retained at least for the periods specified in Directive 2003/94/EC.

## PRODUCTION

**Packaging materials**

15. Specifications and quality control checks should include measures to guard against unintentional unblinding due to changes in appearance between different batches of packaging materials**.**

**Manufacturing operations**

16. During development critical parameters should be identified and in-process controls primarily used to control the process. Provisional production parameters and in-process controls may be deduced from prior experience, including that gained from earlier development work. Careful consideration by key personnel is called for in order to formulate the necessary instructions and to adapt them continually to the experience gained in production. Parameters identified and controlled should be justifiable based on knowledge available at the time.

17. Production processes for investigational medicinal products are not expected to be validated to the extent necessary for routine production but premises and equipment are expected to be qualified. For sterile products, the validation of sterilising processes should be of the same standard as for products authorised for marketing. Likewise, when required, virus inactivation/removal and that of other impurities of biological origin should be demonstrated, to assure the safety of biotechnologically derived

products, by following the scientific principles and techniques defined in the available guidance in this area.

18. Validation of aseptic processes presents special problems when the batch size is small; in these cases the number of units filled may be the maximum number filled in production. If practicable, and otherwise consistent with simulating the process, a larger number of units should be filled with media to provide greater confidence in the results obtained. Filling and sealing is often a manual or semi-automated operation presenting great challenges to sterility so enhanced attention should be given to operator training, and validating the aseptic technique of individual operators.

**Principles applicable to comparator product**

19. If a product is modified, data should be available (e.g. stability, comparative dissolution, bioavailability) to demonstrate that these changes do not significantly alter the original quality characteristics of the product.

20. The expiry date stated for the comparator product in its original packaging might not be applicable to the product where it has been repackaged in a different container that may not offer equivalent protection, or be compatible with the product. A suitable use-by date, taking into account the nature of the product, the characteristics of the container and the storage conditions to which the article may be subjected, should be determined by or on behalf of the sponsor. Such a date should be justified and must not be later than the expiry date of the original package. There should be compatibility of expiry dating and clinical trial duration.

**Blinding operations**

21. Where products are blinded, systems should be in place to ensure that the blind is achieved and maintained while allowing for identification of "blinded" products when necessary, including the batch numbers of the products before the blinding operation. Rapid identification of product should also be possible in an emergency.

**Randomisation code**

22. Procedures should describe the generation, security**,** distribution, handling and retention of any randomisation code used for packaging investigational products, and code-break mechanisms. Appropriate records should be maintained.

**Packaging**

23. During packaging of investigational medicinal products, it may be necessary to handle different products on the same packaging line at the same time. The risk of product mix up must be minimised by using appropriate procedures and/or, specialised equipment as appropriate and relevant staff training.

24. Packaging and labelling of investigational medicinal products are likely to be more complex and more liable to errors (which are also harder to detect) than for marketed products, particularly when "blinded" products with similar appearance are used. Precautions against mis-labelling such as label reconciliation, line clearance, in process control checks by appropriately trained staff should accordingly be intensified.

25. The packaging must ensure that the investigational medicinal product remains in good condition during transport and storage at intermediate destinations. Any opening or tampering of the outer packaging during transport should be readily discernible.

**Labelling**

26. Table 1 summarises the contents of Articles 26-30 that follow. Labelling should comply with the requirements of Directive 2003/94/EC. The following information should be included on labels, unless its absence can be justified, e.g. use of a centralised electronic randomisation system:

    (a) name, address and telephone number of the sponsor, contract research organisation or investigator (the main contact for information on the product, clinical trial and emergency unblinding);

    (b) pharmaceutical dosage form, route of administration, quantity of dosage units, and in the case of open trials, the name/identifier and strength/potency;

    (c) the batch and/or code number to identify the contents and packaging operation;

    (d) a trial reference code allowing identification of the trial, site, investigator and sponsor if not given elsewhere;

    (e) the trial subject identification number/treatment number and where relevant, the visit number;

    (f) the name of the investigator (if not included in (a) or (d));

    (g) directions for use (reference may be made to a leaflet or other explanatory document intended for the trial subject or person administering the product);

    (h) "For clinical trial use only" or similar wording;

    (i) the storage conditions;

    (j) period of use (use-by date, expiry date or re-test date as applicable), in month/year format and in a manner that avoids any ambiguity.

    (k) "keep out of reach of children" except when the product is for use in trials where the product is not taken home by subjects.

27. The address and telephone number of the main contact for information on the product, clinical trial and for emergency unblinding need not appear on the label where the subject has been given a leaflet or card which provides these details and has been instructed to keep this in their possession at all times.

28. Particulars should appear in the official language(s) of the country in which the investigational medicinal product is to be used. The particulars listed in Article 26 should appear on the primary packaging and on the secondary packaging (except for the cases described in Articles 29 and 30). The requirements with respect to the contents of the label on the primary and outer packaging are summarised in Table 1. Other languages may be included.

29. When the product is to be provided to the trial subject or the person administering the medication within a primary package together with secondary packaging that is intended to remain together, and the secondary packaging carries the particulars listed in Paragraph 26, the following information shall be included on the label of the primary package (or any sealed dosing device that contains the primary packaging):

    (a) name of sponsor, contract research organisation or investigator;

    (b) pharmaceutical dosage form, route of administration (may be excluded for oral solid dose forms), quantity of dosage units and in the case of open label trials, the name/identifier and strength/potency;

    (c) batch and/or code number to identify the contents and packaging operation;

    (d) a trial reference code allowing identification of the trial, site, investigator and sponsor if not given elsewhere;

    (e) the trial subject identification number/treatment number and where relevant, the visit number.

30. If the primary packaging takes the form of blister packs or small units such as ampoules on which the particulars required in Paragraph 26 cannot be displayed, secondary packaging should be provided bearing a label with those particulars. The primary packaging should nevertheless contain the following:

    (a) name of sponsor, contract research organisation or investigator;

    (b) route of administration (may be excluded for oral solid dose forms) and in the case of open label trials, the name/identifier and strength/potency;

    (c) batch and/or code number to identify the contents and packaging operation;

    (d) a trial reference code allowing identification of the trial, site, investigator and sponsor if not given elsewhere;

    (e) the trial subject identification number/treatment number and where relevant, the visit number;

31. Symbols or pictograms may be included to clarify certain information mentioned above. Additional information, warnings and/or handling instructions may be displayed.

32. For clinical trials with the characteristics identified in Article 14 of Directive 2001/20/EC, the following particulars should be added to the original container but should not obscure the original labelling:

    i) name of sponsor, contract research organisation or investigator;

    ii) trial reference code allowing identification of the trial site, investigator and trial subject.

33. If it becomes necessary to change the use-by date, an additional label should be affixed to the investigational medicinal product. This additional label should state the new use-by date and repeat the batch number. It may be superimposed on the old use-by date, but for quality control reasons, not on the original batch number. This operation should be

performed at an appropriately authorised manufacturing site. However, when justified, it may be performed at the investigational site by or under the supervision of the clinical trial site pharmacist, or other health care professional in accordance with national regulations. Where this is not possible, it may be performed by the clinical trial monitor(s) who should be appropriately trained. The operation should be performed in accordance with GMP principles, specific and standard operating procedures and under contract, if applicable, and should be checked by a second person. This additional labelling should be properly documented in both the trial documentation and in the batch records.

**QUALITY CONTROL**

34. As processes may not be standardised or fully validated, testing takes on more importance in ensuring that each batch meets its specification.

35. Quality control should be performed in accordance with the Product Specification File and in accordance with the information notified pursuant to Article 9(2) of Directive 2001/20/EC. Verification of the effectiveness of blinding should be performed and recorded.

36. Samples are retained to fulfill two purposes; firstly to provide a sample for analytical testing and secondly to provide a specimen of the finished product. Samples may therefore fall into two categories:

    *Reference sample*: a sample of a batch of starting material, packaging material, product contained in its primary packaging or finished product which is stored for the purpose of being analysed should the need arise. Where stability permits, reference samples from critical intermediate stages (e.g. those requiring analytical testing and release) or intermediates, which are transported outside of the manufacturer's control, should be kept.

    *Retention sample*: a sample of a packaged unit from a batch of finished product for each packaging run/trial period. It is stored for identification purposes. For example, presentation, packaging, labeling, leaflet, batch number, expiry date should the need arise.

    In many instances the reference and retention samples will be presented identically, i.e. as fully packaged units. In such circumstances, reference and retention samples may be regarded as interchangeable.Reference and retention samples of investigational medicinal product, including blinded product should be kept for at least two years after completion or formal discontinuation of the last clinical trial in which the batch was used, whichever period is the longer.

    Consideration should be given to keeping retention samples until the clinical report has been prepared to enable confirmation of product identity in the event of, and as part of an investigation into inconsistent trial results.

37. The storage location of Reference and Retention samples should be defined in a Technical Agreement between the sponsor and manufacturer(s) and should allow timely access by the competent authorities.

*Reference samples* of finished product should be stored within the EEA or in a third country where appropriate arrangements have been made by the Community with the exporting country to ensure that the manufacturer of the investigational medicinal product applies standards of good manufacturing practice at least equivalent to those laid down by the Community. In exceptional circumstances the reference samples of the finished product may be stored by the manufacturer in another third country, in which case this should be justified, and documented in a technical agreement between the sponsor, importer in the EEA and that third country manufacturer.

The reference sample should be of sufficient size to permit the carrying out, on, at least, two occasions, of the full analytical controls on the batch in accordance with the IMP dossier submitted for authorisation to conduct the clinical trial.

In the case of *retention samples*, it is acceptable to store information related to the final packaging as written or electronic records if such records provide sufficient information. In the case of the latter, the system should comply with the requirements of Annex 11.

**RELEASE OF BATCHES**

38. Release of investigational medicinal products (see paragraph 43) should not occur until after the Qualified Person has certified that the requirements of Article 13.3 of Directive 2001/20/EC have been met (see paragraph 39). The Qualified Person should take into account the elements listed in paragraph 40 as appropriate.

39. The duties of the Qualified Person in relation to investigational medicinal products are affected by the different circumstances that can arise and are referred to below. Table 2 summarises the elements that need to be considered for the most common circumstances:

    (a) i) Product manufactured within EU but not subject to an EU marketing authorisation: the duties are laid down in article 13.3(a) of Directive 2001/20/EC.

    (b) ii) Product sourced from the open market within EU in accordance with Article 80(b) of Directive 2001/83/EC and subject to an EU marketing authorisation, regardless of manufacturing origin: the duties are as described above, however, the scope of certification can be limited to assuring that the products are in accordance with the notification/request for authorisation to conduct the trial and any subsequent processing for the purpose of blinding, trial-specific packaging and labelling. The Product Specification File will be similarly restricted in scope (see 9).

    (c) Product imported directly from a 3rd country: the duties are laid down in article 13.3(b) of Directive 2001/20/EC. Where investigational medicinal products are imported from a 3rd country and they are subject to arrangements concluded between the Community and that country, such as a Mutual Recognition Agreement (MRA), equivalent standards of Good Manufacturing Practice apply provided any such agreement is relevant to the product in question. In the absence of an MRA, the Qualified Person should determine that equivalent standards of Good Manufacturing Practice apply through knowledge of the quality system employed at the manufacturer. This knowledge is normally acquired through audit of the manufacturer's quality

systems. In either case, the Qualified Person may then certify on the basis of documentation supplied by the 3rd country manufacturer (see 40).

(d) For imported comparator products where adequate assurance cannot be obtained in order to certify that each batch has been manufactured to equivalent standards of Good Manufacturing Practice, the duty of the Qualified Person is defined in article 13.3(c) of Directive 2001/20/EC.

40. Assessment of each batch for certification prior to release may include as appropriate:

- batch records, including control reports, in-process test reports and release reports demonstrating compliance with the product specification file, the order, protocol and randomisation code. These records should include all deviations or planned changes, and any consequent additional checks or tests, and should be completed and endorsed by the staff authorised to do so according to the quality system;
- production conditions;
- the validation status of facilities, processes and methods;
- examination of finished packs;
- where relevant, the results of any analyses or tests performed after importation;
- stability reports;
- the source and verification of conditions of storage and shipment;
- audit reports concerning the quality system of the manufacturer;
- Documents certifying that the manufacturer is authorised to manufacture investigational medicinal products or comparators for export by the appropriate authorities in the country of export;
- where relevant, regulatory requirements for marketing authorisation, GMP standards applicable and any official verification of GMP compliance;
- all other factors of which the QP is aware that are relevant to the quality of the batch.

The relevance of the above elements is affected by the country of origin of the product, the manufacturer, and the marketed status of the product (with or without a marketing authorisation, in the EU or in a third country) and its phase of development. The sponsor should ensure that the elements taken into account by the qualified person when certifying the batch are consistent with the information notified pursuant to Article 9(2) of Directive 2001/20/EC. See also section 44.

41. Where investigational medicinal products are manufactured and packaged at different sites under the supervision of different Qualified Persons, the recommendations listed in Annex 16 to the GMP Guide should be followed as applicable.

42. Where, permitted in accordance with local regulations, packaging or labelling is carried out at the investigator site by, or under the supervision of a clinical trials pharmacist, or other health care professional as allowed in those regulations, the Qualified Person is not

required to certify the activity in question. The sponsor is nevertheless responsible for ensuring that the activity is adequately documented and carried out in accordance with the principles of GMP and should seek the advice of the Qualified Person in this regard.

## SHIPPING

43. Investigational medicinal products should remain under the control of the sponsor until after completion of a two-step procedure: certification by the Qualified Person; and release by the sponsor for use in a clinical trial following fulfillment of the requirements of Article 9 (Commencement of a clinical trial) of Directive 2001/20/EC. Both steps should be recorded[3] and retained in the relevant trial files held by or on behalf of the sponsor. The Sponsor should ensure that the details set out in the clinical trial application and considered by the Qualified Person are consistent with what is finally accepted by the Competent Authorities. Suitable arrangements to meet this requirement should be established. In practical terms, this can best be achieved through a change control process for the Product Specification File and defined in a Technical Agreement between the QP and the Sponsor.

44. Shipping of investigational products should be conducted according to instructions given by or on behalf of the sponsor in the shipping order.

45. De-coding arrangements should be available to the appropriate responsible personnel before investigational medicinal products are shipped to the investigator site.

46. A detailed inventory of the shipments made by the manufacturer or importer should be maintained. It should particularly mention the addressees' identification.

47. Transfers of investigational medicinal products from one trial site to another should remain the exception. Such transfers should be covered by standard operating procedures. The product history while outside of the control of the manufacturer, through for example, trial monitoring reports and records of storage conditions at the original trial site should be reviewed as part of the assessment of the product's suitability for transfer and the advice of the Qualified person should be sought. The product should be returned to the manufacturer, or another authorised manufacturer, for re-labelling, if necessary, and certification by a Qualified Person. Records should be retained and full traceability ensured.

## COMPLAINTS

48. The conclusions of any investigation carried out in relation to a complaint which could arise from the quality of the product should be discussed between the manufacturer or importer and the sponsor (if different). This should involve the Qualified Person and those responsible for the relevant clinical trial in order to assess any potential impact on the trial, product development and on subjects.

---

[3] A harmonised format for batch certification to facilitate movement between Member States is provided in attachment 3.

## Recalls and returns

### Recalls

49. Procedures for retrieving investigational medicinal products and documenting this retrieval should be agreed by the sponsor, in collaboration with the manufacturer or importer where different. The investigator and monitor need to understand their obligations under the retrieval procedure.

50. The Sponsor should ensure that the supplier of any comparator or other medication to be used in a clinical trial has a system for communicating to the Sponsor the need to recall any product supplied.

### Returns

51. Investigational medicinal products should be returned on agreed conditions defined by the sponsor, specified in approved written procedures.

52. Returned investigational medicinal products should be clearly identified and stored in an appropriately controlled, dedicated area. Inventory records of the returned medicinal products should be kept.

## Destruction

53. The Sponsor is responsible for the destruction of unused and/or returned investigational medicinal products. Investigational medicinal products should therefore not be destroyed without prior written authorisation by the Sponsor.

54. The delivered, used and recovered quantities of product should be recorded, reconciled and verified by or on behalf of the sponsor for each trial site and each trial period. Destruction of unused investigational medicinal products should be carried out for a given trial site or a given trial period only after any discrepancies have been investigated and satisfactorily explained and the reconciliation has been accepted. Recording of destruction operations should be carried out in such a manner that all operations may be accounted for. The records should be kept by the Sponsor.

55. When destruction of investigational medicinal products takes place a dated certificate of, or receipt for destruction, should be provided to the sponsor. These documents should clearly identify, or allow traceability to, the batches and/or patient numbers involved and the actual quantities destroyed.

## TABLE 1: SUMMARY OF LABELLING DETAILS (§26 TO 30)

a) name, address and telephone number of the sponsor, contract research organisation or investigator (the main contact for information on the product, clinical trial and emergency unblinding);

(b) pharmaceutical dosage form, route of administration, quantity of dosage units, and in the case of open trials, the name/identifier and strength/potency;

(c) the batch and/or code number to identify the contents and packaging operation;

(d) a trial reference code allowing identification of the trial, site, investigator and sponsor if not given elsewhere;

(e) the trial subject identification number/treatment number and where relevant, the visit number;

(f) the name of the investigator (if not included in (a) or (d);

(g) directions for use (reference may be made to a leaflet or other explanatory document intended for the trial subject or person administering the product

(h) "for clinical trial use only" or similar wording;

(i) the storage conditions;

(j) period of use (use-by date, expiry date or re-test date as applicable), in month/year format and in a manner that avoids any ambiguity.

(k) "keep out of reach of children" except when the product is for use in trials where the product is not taken home by subjects.

**GENERAL CASE**
For both the primary and secondary packaging (§26)

Particulars a[4] to k

**PRIMARY PACKAGE**
Where primary and secondary packaging remain together throughout (§29)[5]

a[6] b[7] c d e

**PRIMARY PACKAGE**
Blisters or small packaging units (§30)[5]

a[6] b[7,8] c d e

---

[4] The address and telephone number of the main contact for information on the product, clinical trial and for emergency unblinding need not appear on the label where the subject has been given a leaflet or card which provides these details and has been instructed to keep this in their possession at all times (§ 27).

[5] When the outer packaging carries the particulars listed in Article 26.

[6] The address and telephone number of the main contact for information on the product, clinical trial and for emergency unblinding need not be included.

[7] Route of administration may be excluded for oral solid dose forms.

[8] The pharmaceutical dosage form and quantity of dosage units may be omitted.

Commission Européenne, B-1049 Bruxelles / Europese Commissie, B-1049 Brussel – Belgium. Telephone: (32-2) 299 11 11

# Table 2: BATCH RELEASE OF PRODUCTS

| **ELEMENTS TO BE TAKEN INTO ACCOUNT**(3) | **PRODUCT AVAILABLE IN THE EU** | | **PRODUCT IMPORTED FROM THIRD COUNTRIES** | | |
|---|---|---|---|---|---|
| | **Product manufactured in EU without MA** | **Product with MA and available on EU market** | **Product without any EU MA** | **Product with a EU MA** | **Comparator where documentation certifying that each batch has been manufactured in conditions at least equivalent to those laid down in Directive 2003/94/EC cannot be obtained** |
| **BEFORE CLINICAL TRIAL PROCESSING** | | | | | |
| a) Shipping and storage conditions | Yes | | | | |
| b) All relevant factors (1) showing that each batch has been manufactured and released in accordance with:<br>Directive 2003/94/EC, or<br>GMP standards at least equivalent to those laid down in Directive 2003/94/EC. | Yes<br>- | | Yes (2) | | |
| c) Documentation showing that each batch has been released within the EU according to EU GMP requirements (see Directive 2001/83/EC, article 51), or documentation showing that the product is available on the EU market and has been procured in accordance with article 80(b) of Directive 2001/83/EC. | | Yes | | | |
| d) Documentation showing that the product is available on the local market and documentation to establish confidence in the local regulatory requirements for marketing authorisation and release for local use. | | | | | Yes |
| e) Results of all analysis, tests and checks performed to assess the quality of the imported batch according to:<br>the requirements of the MA (see Directive 2001/83/EC, article 51b), or<br>the Product Specification File, the Order, article 9.2 submission to the regulatory authorities. | | | -<br>Yes | Yes<br>- | -<br>Yes |
| Where these analyses and tests are not performed in the EU, this should be justified and the QP must certify that they have been carried out in accordance with GMP standards at least equivalent to those laid down in Directive 2003/94/EC. | | | Yes | Yes | Yes |
| **AFTER CLINICAL TRIAL PROCESSING** | | | | | |
| f) In addition to the assessment before clinical trial processing, all further relevant factors (1) showing that each batch has been processed for the purposes of blinding, trial-specific packaging, labelling and testing in accordance with:<br>Directive 2003/94/EC, or<br>GMP standards at least equivalent to those laid down in Directive 2003/94/EC. | Yes<br>- | | Yes (2) | | |

[illegible]

**ATTACHMENT 3**

[LETTERHEAD OF MANUFACTURER]

## Content of the Batch Certificate

## Referred to in Art. 13.3 Directive 2001/20/EC

(1) Name(s) of product(s)/product identifier(s) as referred to in the clinical trial application, where applicable.

(2) EudraCT No(s) and sponsor protocol code number, when available.

(3) Strength

*Identity (name) and amount per unit dose for all active substance(s) for each IMP (including placebo). The manner in which this information is provided should not unblind the study.*

(4) Dosage form (pharmaceutical form)

(5) Package size (contents of container) and type (e.g. vials, bottles, blisters).

(6) Lot/batch number

(7) Expiry/retest/use by date

(8) Name and address of manufacturer where the Qualified Person issuing the certificate is located.

(9) Manufacturing Authorisation number for the site listed under item 8.

(10) Comments/remarks

(11) Any additional information considered relevant by the QP.

(12) Certification statement.

(13) "I hereby certify that this batch complies with the requirements of Article 13.3 of Directive 2001/20/EC "

(14) Name of the QP signing the certificate

(15) Signature

(16) Date of signature

**<u>Explanatory Note</u>**

Investigational medicinal products may not be used in a clinical trial in a member state of the European Economic Area until the completion of the two-step procedure referred to in section 43 of this Annex. The first step is the certification of each batch by the Qualified Person of the manufacturer or importer that the provisions of Article 13.3(a),

(b) or (c) of Directive 2001/20/EC have been complied with and documented in accordance with Art. 13.4 of the same Directive. According to Directive 2001/20/EC a batch of investigational medicinal product shall not have to undergo further checks in relation to the provisions of article 13.3(a), (b) or (c) of the same directive when it moves between Member States accompanied by batch certification signed by the Qualified Person. In order to facilitate the free movement of investigational medicinal products between Member States the content of these certificates should be in accordance with the above harmonised format. This format may also be used to certify batches destined for use within the Member State of the manufacturer or importer.



Vijiaratnam N, *et al. BMJ Open* 2021; 11:e047993. doi: 10.1136/bmjopen-2020-047993
