# Supplementary material for: Exenatide once weekly over 2 years as a potential disease-modifying treatment for Parkinson’s disease: protocol for a multicentre, randomised, double blind, parallel group, placebo controlled, phase 3 trial: The ‘Exenatide-PD3’ study
Source: BMJ Open. 2021 May 28;11(5):e047993. doi: 10.1136/bmjopen-2020-047993 (PMC8166598; doi:10.1136/bmjopen-2020-047993)
Supplement: Supplementary data [file bmjopen-2020-047993supp002.pdf]

UCL COMPREHENSIVE CLINICAL TRIALS UNIT

NHS University College London Hospitals NHS Foundation Trust

Exenatide-PD3 Site Number: ___ ___ **Exenatide-PD3 Patient Identifiers:**
Screening ID: __ __ - __ __ __ __ __
Trial ID (Exnnn): __ __ __ __ __

Sponsor R&D Number: 18/0320 **Chief Investigator: Professor Tom Foltynie**

Exenatide-PD3 Informed Consent Form

A randomised, double blind, parallel group, placebo controlled, Phase 3 trial of Exenatide once weekly over 2 years as a potential disease modifying treatment for Parkinson's disease.

This Informed Consent Form is intended for consenting patients to take part in the Exenatide-PD3 trial.

	**CONSENT FORM**	Please initial
1	I have read the Exenatide-PD3 information sheet version (X.X)_________dated (DD/MMM/YYYY) _______________ for this trial. I have had the opportunity to consider the information and ask questions that have been answered satisfactorily.	
2	I understand that my participation in the Exenatide-PD3 is voluntary and that I am free to withdraw at any time, without giving any reason and without my medical care or legal rights being affected.	
3	I understand that I may not benefit directly by participating in this trial. However, the research may help others in the future.	
4	I understand that relevant sections of my medical notes and data collected during the trial may be looked at by individuals from the sponsors' office (University College London), regulatory authorities, or from the NHS Trust or drug manufacturer, where it is relevant to my taking part in this research. I understand that these individuals have a duty of confidentiality towards me.	
5	I understand that one of my assessments at five of my visits will be video recorded, stored securely on a GDPR compliant server and securely transferred to Machine Medicines Technologies (MMT) for analysis. I understand that they will be used for quality control purposes, and to help improve PD assessments.	
6	I understand that I am required to attend five of my visits off of my normal PD medication, as stated in the Patient Information Sheet.	

Vijiaratnam N, *et al. BMJ Open* 2021; 11:e047993. doi: 10.1136/bmjopen-2020-047993

7	If the trial research team are unable to contact me at any time during the trial, I agree to the relevant sections of my medical records on NHS Digital (Spine) being accessed to obtain my contact details.	
8	I agree to my General Practitioner (GP) being informed of my participation in the Exenatide-PD3 trial.	
9	I agree that my data gathered in this trial will be stored in a secure facility (with limited access by individual from the sponsor's office (University College London) for 10 years after the completion of the trial as set out by the UK Medicines & Healthcare products Regulatory Agency.	
10	I give consent for my data and blood samples collected as part of the trial to be shared with other researchers and used in other ethically approved research following legal requirements to conceal my identity.	
11	I agree to take part in the Exenatide-PD3 Trial.	
12	I understand that one of my assessments at five of my visits will be recorded using an Electromagnetic Sensor (placed on my hand) and the information collected will be stored securely on a GDPR compliant server and securely transferred to The University Of York for analysis. *(Participating sites only).*	

Participant Name<br>______________________________	Date (DD-MMM-YYYY)<br>_ _/_ _ _/_ _ _ _	Signature<br>____________________________________
Person taking Consent<br>______________________________	Date (DD-MMM-YYYY)<br>_ _/_ _ _/_ _ _ _	Signature<br>____________________________________
Witness (if applicable)<br>______________________________	Date (DD-MMM-YYYY)<br>_ _/_ _ _/_ _ _ _	Signature<br>____________________________________

Once completed, please give 1 copy to the participant, keep 1 copy in the participant's medical records and file the original in the investigator site file.

Vijiaratnam N, *et al. BMJ Open* 2021; 11:e047993. doi: 10.1136/bmjopen-2020-047993
