# Supplementary material for: Exenatide once weekly over 2 years as a potential disease-modifying treatment for Parkinson’s disease: protocol for a multicentre, randomised, double blind, parallel group, placebo controlled, phase 3 trial: The ‘Exenatide-PD3’ study
Source: BMJ Open. 2021 May 28;11(5):e047993. doi: 10.1136/bmjopen-2020-047993 (PMC8166598; doi:10.1136/bmjopen-2020-047993)
Supplement: Author's manuscript [file bmjopen-2020-047993.draft_revisions.pdf]

BMJ Open

BMJ Open is committed to open peer review. As part of this commitment we make the peer review history of every article we publish publicly available.

When an article is published we post the peer reviewers' comments and the authors' responses online. We also post the versions of the paper that were used during peer review. These are the versions that the peer review comments apply to.

The versions of the paper that follow are the versions that were submitted during the peer review process. They are not the versions of record or the final published versions. They should not be cited or distributed as the published version of this manuscript.

BMJ Open is an open access journal and the full, final, typeset and author-corrected version of record of the manuscript is available on our site with no access controls, subscription charges or pay-per-view fees (http://bmjopen.bmj.com).

If you have any questions on BMJ Open's open peer review process please email info.bmjopen@bmj.com

BMJ Open

Exenatide once weekly over 2 years as a potential disease modifying treatment for Parkinson's disease: protocol for a multi-centre, randomised, double blind, parallel group, placebo controlled, Phase 3 trial, The 'Exenatide-PD3' study

Journal:	*BMJ Open*
Manuscript ID	bmjopen-2020-047993
Article Type:	Protocol
Date Submitted by the Author:	17-Dec-2020
Complete List of Authors:	Vijiaratnam, Nirosen; UCL,<br>Girges, Christine; UCL Queen Square Institute of Neurology, Department of Clinical and Movement Neurosciences; National Hospital for Neurology and Neurosurgery, Department of Clinical and Movement Neurosciences<br>Auld, Grace; University College London<br>Chau, Marisa; University College London, Division of Surgery & Interventional Science<br>Maclagan, Kate; UCL, The Comprehensive Clinical Trials Unit<br>King, Alexa; University College London<br>Skene, Simon; University of Surrey, Surrey Clinical Trials Unit<br>Chowdhury, Kashfia; University College London, Comprehensive Clinical Trials Unit<br>Hibbert, Steve; University College London<br>Morris, Huw ; University College London (UCL)<br>Limousin, Patricia; UCL Queen Square Institute of Neurology, Department of Clinical and Movement Neurosciences; National Hospital for Neurology and Neurosurgery, Department of Clinical and Movement Neurosciences<br>Athauda, Dilan; UCL Queen Square Institute of Neurology, Department of Clinical and Movement Neurosciences; National Hospital for Neurology and Neurosurgery, Department of Clinical and Movement Neurosciences<br>Carroll, Camille; University of Plymouth, Faculty of Medicine and Dentistry<br>Hu, Michele; University of Oxford, Nuffield Department of Clinical Neurosciences<br>Silverdale, Monty; University of Manchester, Department of Neurology and Neurosurgery<br>Duncan, Gordon W; NHS Lothian; University of Edinburgh<br>Chaudhuri, Ray; King\'s College London<br>Lo, Christine; University of Oxford<br>Del Din, Silvia; Newcastle University<br>Yarnall, Alison J; Newcastle University<br>Rochester, Lynn; Newcastle University, Institute of Neuroscience<br>Gibson, Rachel<br>Dickson, John; University College London<br>Hunter, Rachael; University College London, Research Dept of Primary Care and Population Health<br>Libri, Vincenzo; National Hospital for Neurology and Neurosurgery;

	Leonard Wolfson Experimental Neurology Centre<br>Foltynie, Thomas; UCL Queen Square Institute of Neurology, Department of Clinical and Movement Neurosciences; National Hospital for Neurology and Neurosurgery, Department of Clinical and Movement Neurosciences
Keywords:	Parkinson-s disease < NEUROLOGY, PUBLIC HEALTH, QUALITATIVE RESEARCH

SCHOLARONE™
Manuscripts

I, the Submitting Author has the right to grant and does grant on behalf of all authors of the Work (as defined in the below author licence), an exclusive licence and/or a non-exclusive licence for contributions from authors who are: i) UK Crown employees; ii) where BMJ has agreed a CC-BY licence shall apply, and/or iii) in accordance with the terms applicable for US Federal Government officers or employees acting as part of their official duties; on a worldwide, perpetual, irrevocable, royalty-free basis to BMJ Publishing Group Ltd ("BMJ") its licensees and where the relevant Journal is co-owned by BMJ to the co-owners of the Journal, to publish the Work in this journal and any other BMJ products and to exploit all rights, as set out in our licence.

The Submitting Author accepts and understands that any supply made under these terms is made by BMJ to the Submitting Author unless you are acting as an employee on behalf of your employer or a postgraduate student of an affiliated institution which is paying any applicable article publishing charge ("APC") for Open Access articles. Where the Submitting Author wishes to make the Work available on an Open Access basis (and intends to pay the relevant APC), the terms of reuse of such Open Access shall be governed by a Creative Commons licence – details of these licences and which Creative Commons licence will apply to this Work are set out in our licence referred to above.

Other than as permitted in any relevant BMJ Author's Self Archiving Policies, I confirm this Work has not been accepted for publication elsewhere, is not being considered for publication elsewhere and does not duplicate material already published. I confirm all authors consent to publication of this Work and authorise the granting of this licence.

For peer review only

Exenatide once weekly over 2 years as a potential disease modifying treatment for Parkinson's disease: protocol for a multi-centre, randomised, double blind, parallel group, placebo controlled, Phase 3 trial, The 'Exenatide-PD3' study

Nirosen Vijiaratnam[1,2], Christine Girges[1,2], Grace Auld[3], Marisa Chau[3], Kate MacLagan[3], Alexa King[3], Simon Skene[4], Kashfia Chowdhury[3], Steve Hibbert[3], Huw Morris[1,2] Patricia Limousin[1,2], Dilan Athauda[1,2], Camille B. Carroll[5,6], Michele T. Hu[7-9], Monty Silverdale[10], Gordon W Duncan[16,17] Ray Chaudhuri[14], Christine Lo[8], Silvia Del Din[15], Alison J Yarnall[15,18], Lynn Rochester[15], Rachel Gibson, John Dickson[11], Rachael Hunter[12], Vincenzo Libri[2,13] & Thomas Foltynie[1,2]

1. Department of Clinical and Movement Neurosciences, UCL Queen Square Institute of Neurology, London, UK.

2. The National Hospital for Neurology and Neurosurgery, Queen Square, London, UK.

3. Comprehensive Clinical Trials Unit, University College London, London, UK

4. Department of Clinical and Experimental Medicine, University of Surrey, Guildford, UK.

5. Applied Parkinson's Research Group, University of Plymouth, Faculty of Health, Plymouth, Devon, United Kingdom.

6. University Hospitals Plymouth NHS Trust, Plymouth, Devon, United Kingdom.

7. Nuffield Department of Clinical Neurosciences, Division of Clinical Neurology, University of Oxford, Oxford, UK.

8. Oxford Parkinson's Disease Centre, University of Oxford, Oxford, UK.

9. Department of Clinical Neurology, Oxford University Hospitals NHS Foundation Trust, Oxford, UK.

10. Department of Neurology and Neurosurgery, Salford Royal NHS Foundation Trust, Manchester Academic Health Science Centre, University of Manchester, Greater Manchester, UK.

11. Institute of Nuclear Medicine, University College London, London, UK.

12. Research Department of Primary Care and Population Health, Royal Free Medical School, University College London, Royal Free Campus, Rowland Hill Street, London, NW3 2PF, United Kingdom.

13. National Institute for Health Research, University College London Hospitals NHS Foundation Trust, Clinical Research Facility and Leonard Wolfson Experimental Neurology Centre.

14. Parkinson Foundation International Centre of Excellence, King's College Hospital and King's College London

15. Translational and Clinical Research Institute, Faculty of Medical Sciences, Newcastle University, Newcastle upon Tyne

16. Western General Hospital, NHS Lothian, Edinburgh, UK

17. University of Edinburgh, Edinburgh, UK

18. Newcastle upon Tyne NHS Foundation Trust, Newcastle, UK

For peer review only

Corresponding author:

Prof Thomas Foltynie

Department of Clinical and Movement Neurosciences,

UCL Queen Square Institute of Neurology,

London, UK.

Email: t.foltynie@ucl.ac.uk

Trial Registration Number: Clinicaltrials.gov identifier: NCT04232969, ISRCTN14552789

Key Words:

Exenatide; Parkinson's disease; randomised control trial.

Abstract

Introduction

Parkinson's disease (PD) is a common neurodegenerative disorder with substantial morbidity. No disease modifying treatments currently exist. The glucagon like peptide-1 receptor agonist exenatide has been associated in single centre studies with reduced motor deterioration over 1 year. The aim of this multicentre UK trial is to confirm whether these previous positive results are maintained in a larger number of participants over two years and if effects accumulate with prolonged drug exposure.

Methods and analysis

This is a phase 3, multi-center, double-blind, randomised, placebo-controlled trial of exenatide at a dose of 2 mg weekly in 200 participants with mild to moderate PD. Treatment duration is 96 weeks. Randomisation is 1:1, drug to placebo. Assessments are performed at baseline, week 12, 24, 36, 48, 60, 72, 84, and 96 weeks.

The primary outcome is the comparison of MDS-UPDRS part 3 motor sub-score in the practically defined OFF medication state at 96 weeks between participants according to treatment allocation. Secondary outcomes will compare the change between groups among other motor, non-motor and cognitive scores. The primary outcome will be reported using descriptive statistics and comparisons between treatment groups using a mixed model, adjusting for baseline scores. Secondary outcomes will be summarised between treatment groups using summary statistics and appropriate statistical tests to assess for significant differences.

Ethics and dissemination

This trial has been approved by the Research Ethics Committee and the Health Research Authority. Results will be disseminated in peer-reviewed journals, presented at scientific meetings and to patients in lay-summary format.

Article Summary

Strengths and Limitations of this Study

For peer review only

- This is the protocol for the first phase 3 double-blind, randomised, placebo controlled trial of Exenatide in Parkinson's disease (PD).
- This study uses novel secondary outcome measures in sub-studies (cerebrospinal fluid analysis, dopamine transporter imaging, and digital technology measurement devices) which should provide a more sensitive and comprehensive assessment of potential disease modification.
- Although the 2 year follow-up period should provide a more definitive signal on disease modification this will take longer to report findings and has risks for long-term patient retention

Introduction

Parkinson's disease (PD) is the second most common neurodegenerative disease affecting over 10 million people worldwide and its prevalence is increasing [1]. Symptomatic treatments are available and mainly focus on dopamine replacement strategies [2,3]. Such therapies provide improvements in the core motor features of PD: tremor, limb rigidity and slowness of movement (bradykinesia) [4]. These symptomatic treatments do not impact on the progressive nature of the disease nor the majority of the non-motor symptoms (NMS). Moreover, with time, some patients will develop dopamine-refractory gait and balance problems leading to falls and risk of fractures; speech and swallowing problems leading to difficulty in communication and aspiration pneumonia;, cognitive impairment, visual hallucinations and dementia with mounting care needs.[3,4]. These complications result in increased dependence, care giver strain, need for 24 hour care and death. Therefore, Parkinson's disease is a growing problem for individuals, healthcare and society making the development of disease modifying treatments imperative.

Exenatide (Exendin-4) is a licensed and effective treatment for patients with type 2 diabetes mellitus (T2DM) [5]. It is an agonist for the Glucagon-like peptide 1 (GLP-1) receptor and in the presence of elevated blood glucose stimulates insulin release. It also increases pancreatic beta islet cell mass and reduces apoptosis. Exenatide has been the subject of multiple phase 3 trials in patients with type 2 diabetes and was granted a license for the treatment of type 2 diabetes in 2006 [5].

In parallel with the confirmation of the beneficial effects of exenatide on glucose control, laboratory work has showed that exenatide has beneficial effects on neurons in vitro [6]. Exenatide induces neurite outgrowth, promotes neuronal differentiation and rescues degenerating neuronal cells while also reversing neurotoxin induced damage in animal models [6,7]. These neurotrophic properties have sparked interest regarding its potential use as a neurodegenerative disease modifying agent [8,9].

The specific relevance of exenatide to PD has also been extensively evaluated. Exenatide has been shown to increase transcription of tyrosine hydroxylase (TH) (the rate limiting enzyme in dopamine synthesis) in brainstem catecholaminergic neurons [10]. Furthermore, stimulation of GLP-1 receptors may have beneficial effects on the neurodegenerative processes of PD through downstream cellular pathways [6,11]. These findings are further supported by a recent study suggesting a reduced future risk of developing PD in T2DM patients treated with GLP-1 agents [12].

To investigate the potential effects of exenatide in patients with PD, an investigator-initiated pilot trial was undertaken [13]. This open label, parallel group, randomised controlled trial evaluated the tolerability of exenatide (Byetta 10mcg twice daily) in 45 patients with moderately severe PD (Hoehn and Yahr stage of less than 2.5) over an exposure period of 48 weeks with a subsequent washout

For peer review only

period of 12 weeks. This showed an advantage of 4.9 points in in the Movement Disorders Society Unified Parkinson's Disease Rating Scale (MDS-UPDRS) part 3 (motor sub-score) in exenatide treated patients at 12 months which persisted even after a 12 week washout period. Clinically important differences in cognition were also noted. Serial DaTscan imaging showed no progression between baseline and 48 weeks in the exenatide treated patients [13].

A further Phase 2 double blind randomised controlled trial evaluating the effects of exenatide in 60 patients with PD has subsequently been performed [14]. Patients were randomised to self-injection of a long acting form of exenatide, (Bydureon 2mg) once weekly, or matched placebo for 48 weeks. Detailed assessments every 12 weeks for the duration of the treatment and a further assessment at the 60 week time point to explore any lasting effects following washout of the trial medication were performed. Patients receiving exenatide had a mean 3.5 point advantage in their MDS-UPDRS part 3 OFF medication scores compared to patients receiving placebo at the 60 week timepoint. Biological specimens collected from trial participants confirmed changes according to treatment with exenatide in downstream cellular effector pathways [15].

The current trial objective (Box 1) is to confirm or refute whether the previous positive results can be reproduced in a multicentre trial design, including a larger number of participants evaluated over twice as long a period as previously. An important secondary objective is to explore if positive effects seen after 48 weeks of exenatide exposure remain static or increase in amplitude by the 96-week time point. The hypothesis is that exenatide will be associated with reduced MDS-UPDRS part 3 scores at the 96-week time-point. The overriding priority for this trial is to provide evidence to support or refute any signal of efficacy of exenatide in PD, and thus provide the justification for rapid further investment in this drug if appropriate. In parallel with this, is the aim to explore whether any biological effect(s) of exenatide, relevant to PD, are purely symptomatic effects as opposed to disease modifying effects.

For peer review only

Box 1 Trial Objectives

Primary

- Compare the effectiveness of exenatide once weekly versus placebo on the MDS-UPDRS part 3 motor sub-score in the "practically defined OFF medication state" in patients with PD (Change in the MDS-UPDRS part 3 score reflects accumulation of motor deficit and therefore is a measure of PD motor progression)

Secondary

- Compare differences at 48 and 96 weeks between the exenatide and placebo trial arms in:
  - MDS-UPDRS part 1, 2, 3 and 4 ON medication scores
  - Timed Walk assessment ON and OFF medication
  - Montreal Cognitive Assessment (MoCA)
  - Safety and tolerability of exenatide as indicated by changes in vital signs, weight, clinical laboratory measures and adverse events
  - Patient Health Questionnaire (PHQ-9)
  - Unified Dyskinesia Rating Scale (UDysRS)
  - Parkinson's Disease 39 item Quality of Life questionnaire (PDQ-39)
  - Levodopa equivalent doses
  - 3 day Hauser diary of PD state (Time-On, Off, Non troublesome Dyskinesia, Troublesome dyskinesia, Asleep)

- Compare differences in total values over 96 weeks between the exenatide and placebo trial arms in:
  - Health and social care resource use on the modified Client Service Receipt Inventory (CSRI)
  - Health and social care costs
  - Paid and unpaid carer costs
  - Quality adjusted life years (QALYs) calculated using the EQ-5D-5L tariff adjusting for baseline.

- Compare differences between scores at 48 and 96 weeks between the exenatide and placebo trial arms in:
  - MDS-UPDRS part 3 Motor subsection OFF medication score

Exploratory

Compare differences between slopes at pre-specified periods between exenatide and placebo trial arms for key outcomes to investigate whether exenatide can be considered disease modifying.

Methods

This trial protocol was designed using the University College London (UCL) Comprehensive Clinical Trials Unit (CCTU) Protocol template. The trial is sponsored by UCL and coordinated by the CCTU. The protocol was designed to provide information about procedures for entering participants into the trial, and sufficient detail to enable: an understanding of the background, rationale, objectives, trial population, intervention, methods, statistical analyses, ethical considerations, dissemination plans and administration of the trial; replication of key aspects of trial methods and conduct; and appraisal of the trial's scientific and ethical rigour from the time of ethics approval through to dissemination of the results**.** All stake holders (research team, sponsor, CCTU and oversight committees) were involved in the design and approval of the protocol. A particular emphasis was given to patient input in the trial design. This patient and public involvement (PPI) approach has proven to be of value in other studies [16] and was harnessed to improve the overall study design. A focus group meeting with patients was organized in the protocol design stages to obtain feedback from patients which led to a number of amendments prior to submission, including the maximum overall trial duration of 96 weeks, and the use of OFF-medication assessments. Two PPI representatives will serve on the trial steering committee (TSC) and will continue to provide regular input throughout recruitment. Patients will also be provided access to the trial website and a link to the protocol and patient information sheets (PIS) on request and will be given the opportunity to continue to provide comments and contact researchers to further discuss their input. The INCLUDE guidance [17] is an NIHR led initiative to improve inclusion of under-served groups. The design of the trial is mindful of the value of the steps outlined in this initiative and aims to incorporate its recommendations into overall trial recruitment with the overarching aim of providing better access and quality care to under-served patient groups.

Patient and Public Involvement:

In the development of this protocol, a formal meeting was help with 6 patients with PD, hosted by the Cure Parkinson's Trust. The aims and the objectives of the trial were discussed including the importance of distinguishing between symptomatic and disease modifying effects of exenatide. Patient feedback was clear that a 2 year period would be the maximal acceptable duration of self-administration of placebo, therefore the trial duration was reduced from the original planned 3 year duration to 96 weeks. The use of weekly self-administered injections, and attendance in the off-medication state to assess PD severity was discussed in detail and considered acceptable. The recruitment strategy has used the patient networks of the Cure Parkinson's Trust and Parkinson's UK to increase the awareness of the trial. Patients and patient representatives are included in the Trial Steering committee. At the end of the study, all participants will be notified of their randomisation allocation and of the main study results. The results will be presented at meetings convened for patient groups and published in open access peer reviewed publications.

Trial design

This is a simple parallel group multicentre phase 3, double-blind, randomised, placebo-controlled trial. This will include a 96-week exposure period. Detailed evaluations of all participants will take place at screening, baseline, 24, 48, 72 and 96 weeks (Figure 1). Participants will also attend on a 12

For peer review only

weekly basis to collect supplies of IMP. Participants will be randomly allocated to receive either Exenatide extended release 2mg subcutaneous injection (Bydureon) once weekly for 96 weeks n=100, or Exenatide extended release placebo subcutaneous injection once weekly for 96 weeks n=100. In addition, participants will be randomised using a minimisation algorithm (with a random element incorporated) balancing by research site, participants with greater (Hoehn & Yahr stage 2.5) or lesser (Hoehn & Yahr stage 2.0 or less) PD severity (in the ON medication state), and participation in the sub-studies (Remote Monitoring, Imaging, or not participating).

Participants & recruitment

Patients are eligible for screening if they have a clinical diagnosis of PD. The Queen Square brain bank criteria[18] can be also be used to assist in the diagnosis, however, this is not a formal inclusion criterion, and the relevance of a positive family history of PD, or a confirmed genetic basis for an individual's symptoms will be evaluated in the context of other clinical features in determining diagnosis and eligibility. Key inclusion and exclusion criteria are summarised in Box 2.

Participants will typically be recruited through specialist movement disorders clinics at trial sites. The trial will be advertised online by the Parkinson's UK website, the Cure Parkinson's Trust and the NIHR Clinical Research Network websites and will be registered on ClinicalTrials.gov and the ISRCTN registry. Trial advertisements will direct participants to contact teams in order to be provided with a patient information sheet and a reply slip to confirm ongoing interest and to organise a pre-screening telephone call to discuss eligibility and suitability for the study. It is anticipated that recruitment will be completed from six UK sites (National Hospital for Neurology and Neurosurgery (Queen Square, London), King's College Hospital NHS Foundation Trust (London), Oxford University Hospitals NHS Foundation Trust (Oxford), Derriford University Hospital (Plymouth), Salford Royal Hospital (Manchester) and Western General Hospital & Royal Infirmary of Edinburgh (Edinburgh). Recruitment rates will be carefully monitored throughout the trial to inform on the total number of sites required to ensure final recruitment milestones will be reached. If needed, further sites will be set up. All patient assessments will be performed at hospitals in the UK, once sites have confirmed capacity and capability and a site initiation visit has been performed.

For peer review only

Box 2 Key inclusion and exclusion criteria for study

Key inclusion criteria

- Diagnosis of Parkinson's disease PD based on review of the participant's clinical history, examination findings and response to PD medications. The Queen Square brain bank criteria [18] can be also be used to assist the diagnosis, however this is not a formal inclusion criterion. The relevance of a positive family history of PD, or a confirmed genetic basis for an individual's symptoms will be evaluated in the context of other clinical features in determining diagnosis and eligibility.
- Hoehn and Yahr stage ≤2.5 in the ON medication state. This implies that all patients will be mobile without assistance during their best "ON" medication periods.
- Between 25 and 80 years of age.
- On dopaminergic treatment for at least 4 weeks before enrolment. All participants must have had previous or ongoing exposure to dopaminergic treatment either as L-dopa or a dopamine agonist. If L-dopa has been stopped due to side effects or lack of response, the local PI should further confirm that the participant has clinical symptoms and signs and/or radiological investigations consistent with a diagnosis of Parkinson's disease.
- Ability to self-administer, or to arrange carer administration of trial medication.
- Documented informed consent to participate.

Key exclusion criteria

- Diagnosis or suspicion of other cause for Parkinsonism. Patients with clinical features indicating a diagnosis of Progressive Supranuclear Palsy, Multiple Systems Atrophy, Drug induced Parkinsonism, Dystonic tremor or Essential tremor will not be recruited.
- Patients unable to attend the clinic visits in the practically defined OFF medication state.
- Body mass index <18.5. (Exenatide is known to cause weight loss therefore individuals that may not tolerate further weight loss will not be recruited).
- Known abnormality on CT or MRI brain imaging considered likely to compromise compliance with trial protocol.
- Significant cognitive impairment defined by a score <21 on the Montreal Cognitive Assessment.
- Concurrent severe depression defined by a score ≥16 on the Patient Health Questionnaire (PHQ-9)
- Prior intra-cerebral surgical intervention for Parkinson's disease. Patients who have previously undergone Deep Brain Stimulation, intra-cerebral administration of growth factors, gene therapy or cell therapies will not be eligible.
- Previous participation in one of the following Parkinson's disease trials (Biogen SPARK trial, Prothena Pasadena trial, Sanofi Genzyme MOVES-PD trial, UDCA-PD UP Study or any other trial still considered to involve a potentially PD modifying agent). In the event of any uncertainty, the Chief Investigator will discuss the relevance of exposure to any other specific trials/experimental agents with the local Principal Investigator before recruitment eligibility is confirmed.
- Participation in another clinical trial of a device, drug or surgical treatment within the last 30 days.
- Previous exposure to exenatide.
- Impaired renal function with creatinine clearance <50ml/min.
- History of pancreatitis. Screening serum amylase value must fall within laboratory normal range +/- 50%.
- Type 1 or Type 2 Diabetes mellitus.
- Severe gastrointestinal disease (e.g. gastroparesis)
- Hyperlipidaemia. A lipid profile will be tested at the screening visit. Cholesterol or Triglyceride levels greater than 2 x the upper limit of normal will raise suspicion of a familial or acquired hyperlipidaemia and will prompt referral to a relevant specialist for investigation and treatment.
- History or family history of medullary thyroid cancer (MTC). Undiagnosed neck lump, hoarse voice or difficulty swallowing (not attributable to PD diagnosis).
- Multiple endocrine neoplasia 2 (MEN2) syndrome.
- Hypersensitivity to any of exenatide's excipients.
- Females that are pregnant or breast feeding. There are no safety data regarding exenatide use in pregnancy.
- Women of child bearing potential who are unwilling or unable to use an acceptable method to avoid pregnancy for the entire trial period and up to 3 months after the last dose of trial medication. Female participants who are able to become pregnant (defined as women of child-bearing potential) will undergo a pregnancy test prior to randomisation and will be asked at each visit to confirm regular use of an effective method of contraception
- Participants who lack the capacity to give informed consent.
- Any medical or psychiatric condition or previous conventional/experimental treatment which in the investigator's opinion compromises the potential participant's ability to participate.

For peer review only - http://bmjopen.bmj.com/site/about/guidelines.xhtml

Outcomes

Safety monitoring

Safety and tolerability of exenatide as indicated by changes in vital signs, weight, clinical laboratory measures and adverse events (AEs) will be recorded and monitored throughout. Each patient will have their pulse, blood pressure and weight documented at screening and at each follow up visit. Exenatide is known to cause weight loss. Participants' height will be recorded at screening to enable calculation of body mass index. At each visit, participants are asked to report any AEs that have occurred since the previous visit. AEs may also be detected by the study team reviewing the patient or through notification by the participant's primary care physician. All AEs will be assessed by a study doctor for their severity, likely relationship to study drug and required action by a study doctor not involved in the blinded assessment of the patient. All SAEs will be recorded and reported to the sponsor regardless of relation to trial treatment. Any suspected unexpected serious adverse reactions will be reported to the sponsor immediately to allow facilitation of unblinding as necessary. All AEs reported will be reviewed by the trial management group, trial steering group and monitored by an independent data monitoring committee. Unblinding requests from other clinicians responsible for a patient's care will be handled by the principal investigator (PI) at each site. The PI at each site may also choose to unblind a participant in response to reported AEs as they are reported, if judged to be clinically necessary.

Primary Outcome

The ***MDS-UPDRS part 3 motor*** OFF medication score is a widely accepted measure of the motor disability of PD. The scale is performed in the ON medication state and in the practically defined OFF medication state. This is defined as the score obtained in a patient who has withheld all short acting conventional PD medications for at least 8 hours and all long acting conventional PD medications for at least 36 hours. Comparison of MDS-UPDRS part 3 motor sub-score in the practically defined OFF medication state at 96 weeks between participants according to treatment allocation and adjusted for baseline will be the primary outcome. The scores for these assessments will be collected and recorded by trained clinical trial personnel (if possible, the same person will rate these assessments at each site to minimise inter-rater variability). With consent, these assessments will be video recorded as part of a MDS-UPDRS automated scoring sub study though the availability of these videos will also enable repeated independent scoring to be performed if there are concerns raised about data quality from a specific site/rater.

Secondary Outcomes

Comparisons at 48 and 96 weeks between participants according to treatment allocation will also be performed for each of the secondary outcomes listed below.

The ***MDS-UPDRS part 3 motor score in the practically defined OFF medication state*** at 48 weeks. Whereas the analysis of the 96-week scores according to randomisation group will represent the primary outcome for this trial, differences emerging at 48 weeks and also the difference between scores at 48 weeks and 96 weeks will be important secondary outcomes.

MDS-UPDRS part 1, 2, 3 and 4 ON medication scores. Part 3 of the MDS-UPDRS as well as the other elements (Part 1,2 & 4) of the scale will also be evaluated in the presence of conventional PD medication (ON state) to evaluate any change in some of the non-motor symptoms of PD, activities of daily living and the complications of chronic PD treatment.

For peer review only

Montreal Cognitive assessment (MoCA). This scale is a validated global measure of cognitive ability. This will be assessed in the ON medication state.

Timed Tests. Participants will be asked to perform a ***Sit-stand-walk timed test*** in both the OFF medication and ON medication state. The timed Sit-stand-walk test will incorporate time taken from seated position to stand and walk 10 metres, turn and return to original seated position.

Unified dyskinesia rating scale (UDysRS). This is considered to be the most useful and objective way of quantifying dyskinesia severity. This will be assessed in the ON medication state.

Patient Health Questionnaire-9 (PHQ-9). This scale allows for self-quantification of depression severity. This will be assessed in the ON medication state.

Non-motor symptom scale (NMSS). This validated scale is a tool to collect data on the frequency and severity of 30 non-motor symptoms sometimes experienced by PD patients. This will be assessed in the ON medication state.

The Parkinson's Disease Questionnaire (PDQ-39). This is the standard disease specific measure of quality of life in PD comprising 39 questions. It has been extensively validated in previous studies.

Levodopa equivalent dose (LED). To facilitate comparisons between patients taking different regimes of conventional PD medications, a set of conversion factors have been used to convert each of the commonly used PD medications to an LED of each of their medications can then be summed for inter-patient / inter-group comparisons.[19]

EQ-5D-5L. This is a simple, 5 question form and visual analogue scale that allows calculation of quality adjusted life years (QALY) to enable health economic analyses to be performed.

The Client Service Receipt Inventory (CSRI). Health and social care resource use. Self-completed health care, social care and paid/unpaid carer resource use questionnaire asking about primary and secondary care resource use relevant to Parkinson's and impact on carers in the past 6 months.

3-day Hauser diary of PD state (Time- On, Off, Troublesome Dyskinesia, Non-troublesome dyskinesia, Asleep). Diary data allows quantification of the amount of time during a 3-day period that patients spend in the varying states of movement ability.

Ancillary Studies

There are four optional sub-studies linked to the main trial:

1. Genetics sub-study: To try to identify genetic markers that may be associated with subtypes of PD or variation in treatment responsiveness.

2. Cerebrospinal Fluid (CSF) sub-study: To determine whether any CSF changes associated with PD are influenced by exposure to exenatide. These may include alpha synuclein monomers or oligomers, neuroinflammatory markers, and exosomal contents.

3. Remote Monitoring of PD Symptoms sub-study: To help determine whether measurement of PD symptoms using digital technology may be a more sensitive measure of change with active drug versus placebo compared to the MDS UPDRS 3 in the OFF and ON medication states. This will form two separate measurements comprising 1) home-based smartphone and 2) real-world gait/walking activity monitoring.[20],[21],[22] This aims to generate precision data, providing person-specific distributions of outcomes and may be able to better delineate baseline clinical features.

For peer review only

4. DaTSCAN (Imaging sub-study): To determine if change in dopamine transporter availability in the caudate and putaminal nuclei as measured by quantitative DaTSCAN signal is influenced by exposure to exenatide compared to placebo.

Visits

The overall progression of assessments are summarised in figure 1. While we expect to undertake all assessments in respective clinical units, provision has been made in line with INCLUDE guidance for the possibility of home visit assessments to be performed when patient specific situations (e.g. inability to travel due to coronavirus restrictions, worsening 'OFF' state over progression of trial) necessitate this. We hope that this provision will aid overall trial retention while enhancing recruitment of patients from typically less well represented demographics (e.g. rural geographic regions, patients lacking private travel facilities).

Screening Visit

Written informed consent to enter and be randomised into the trial will be obtained from participants, after explanation of the aims, methods, benefits and potential hazards of the trial and before any trial-specific procedures are performed or any blood is taken for the trial. Patients will be screened using the history of their Parkinson's disease, supported by any available clinical correspondence according to usual standard of care.

The collection of the following scales will evaluate patient eligibility: MoCA, PHQ-9, as well as blood tests (full blood count, urea & electrolytes, creatinine, liver function tests, HbA1C, C-peptide, coagulation, serum amylase, thyroid function tests, blood glucose, insulin and lipid profile, and a pregnancy test for women of child bearing potential). Tests can be repeated between screening and baseline visits, if required to confirm eligibility.

Patients recruited to the DATSCAN substudy will have imaging performing prior to their baseline visit.

Baseline visit and randomisation

Previously defined primary and secondary outcome measures will be performed in the 'ON' and 'OFF' states as outlined below. Patients' LED will be noted. Randomisation to either exenatide or placebo will be administered using a centralised, web-based system (www.sealedenvelope.com). All assessments related to sub-studies will also be performed prior to trial medication administration.

Assessment Procedures

After the Screening Visit, the named site clinical staff member will call the participant to remind them of the need to stop taking their regular PD medication prior to their next trial visit and to attend in a fasted state (prior to visits 2, 4, 6, 8, 10). The MDS-UPDRS part 3 and Timed Walk assessments will be initially performed in the OFF state. This assessment in both the 'OFF' and 'ON' states will be performed with video recording to facilitate the possibility of a re-review if necessary. Remote monitoring assessments will be conducted at this point at selected sites in patients consenting to participate in this sub-study. While waiting for medications to work, participants will self-complete the MDS-UPDRS parts 1, 2 and 4, PDQ-39, EQ-5D-5L and CSRI. The MDS-UPDRS part 3 and Timed Walk assessments will be repeated 1 hour after the participant has taken their routine medications - the ON medication state. After completion of the MDS-UPDRS and Timed Walk assessments in the ON medication state, each participant will be assessed using the MoCA, NMS scale, UDysRS and PHQ-9. This will occur in alternate post-randomisation assessments (at visits 2, 4,

For peer review only

6, 8, 10). At selected centres participants in the CSF sub-study will have a CSF sample taken via lumbar puncture. Ten weeks after the last trial medication administration, a staff member will call the participant to collect details of any adverse events that have occurred after the participant stops taking the trial medication. Participants will complete the 3 Day Hauser Diary prior to visits 2, 6 and 10 and return the diary back to the research team at the respective study visits. At each of the visits 2, 4, 6, 8, and 10, a blood sample will be collected and processed for storage for future analysis.

The DaTSCAN imaging sub-study will be performed at the UCLH site on all consenting sub-study participants; scans will be performed prior to visit 2 and after visit 10.

The option for performing a remote assessment will be provided to patients for safety monitoring visits in view of the coronavirus pandemic.

Intervention

Each dose of Exenatide 2 mg (powder and solvent for prolonged release, suspension for injection, prefilled pen) is supplied as a single use injection pen for subcutaneous administration by the patient on a weekly basis. The placebo (inactive powder and solvent for prolonged release, suspension for injection, prefilled pen) is supplied as an identical injection pen for subcutaneous administration by the patient on a weekly basis. The trial medication will be refrigerated and stored at 2-8°C. Both exenatide and placebo will be supplied by AstraZeneca as unlabelled prefilled pens in bulk and in accordance with Good Manufacturing Practice (GMP). Labelling, packaging and release of packed trial medications will be managed by the Sponsor's contracted company following GMP. The labels will be prepared in accordance with GMP Annex 13 requirements for labelling and local regulatory guidelines. The trial medications will be released ahead of trial use.

Site trial staff will be trained on the use of exenatide using an online teaching video, accompanying product literature and the Investigator's Brochure (IB). Patients will be taught how to perform the subcutaneous injections by the clinical trial team using the online video, demonstration packs and written literature. They will be told about common adverse reactions previously reported e.g. nausea, vomiting, diarrhoea and weight loss by the clinical trial team, and will be advised on the processes for safety reporting. In the event that exenatide injections will be administered by care-givers (e.g. spouse), their willingness to perform this will be documented and they will be trained using the online teaching video. It will also be ascertained that the care-giver either lives with the PD patient or confirms their willingness to meet with the PD participant on a weekly basis to administer the injections for the 96 week period of the trial.

Patients who meet eligibility criteria at the screening visit will be randomly assigned to receive 96 weeks of double-blind treatment with either exenatide or placebo (2mg once weekly) in a 1:1 ratio. The first dose will be administered by the patient in clinic following injection training and subsequent injections will be at home. Injections will be self-administered by the participants, or administered by their carer, into the participants' abdomen, arm, thigh or buttocks every 7 days. Participants will be provided with a link to the injection pen training video and a REC approved injection administration training sheet.

Sample Size

For peer review only

The sample size is based on the detectable effect size (primary outcome is the MDS-UPDRS motor subsection in the OFF medication state) for a two-arm (exenatide versus placebo) parallel-group trial design. The calculations assume a common standard deviation of 13.5, and a correlation of 0.70 between baseline and follow up MDS-UPDRS measurements. These estimates are reasonable based on data from the previous Exenatide-PD trial[14] On this basis, 160 evaluable participants divided equally between the 2 groups is sufficient to detect a difference of 5.0 MDS-UPDRS part 3 points in the OFF medication state between the 2 groups adjusting for baseline MDS-UPDRS part 3 OFF scores, with 90% power and at a significance level of 0.05. Assuming 20% attrition (withdrawal/loss to follow up), 200 participants will be recruited. Participants who withdraw from the trial will not be replaced. Participants who withdraw from trial treatment should remain in the trial for the purpose of follow-up and data analysis. This effect size is a reasonable expectation based on the previously collected pilot data and would represent a clear demonstration of the efficacy of exenatide on the motor severity of PD.

It is also anticipated that the difference in scores in the ON medication state will be greater at 96 weeks than at the earlier time points. The expected rate of change in PD severity in the first 5 years after PD diagnosis in the ON medication state is 1 MDS-UPDRS part 3 point per year. A predicted advantage of 2 points in ON scores over 96 weeks would thus equate to an advantage in the rate of disease progression above and beyond that achievable with conventional dopaminergic medication and would be a further clear signal that continued use of exenatide is consistent with not only long-term disease modifying effects, but even demonstration of a small change of 2.5 points in the MDS-UPDRS motor score would constitute a clinically important difference[23] and potentially an advantage in day to day functional impairment and overall improvement in quality of life in the short term.

Statistical Analysis

A full statistical analysis plan (SAP) will be written and approved by the Trial Steering Committee prior to database lock. All analyses will be undertaken according to a modified intention-to-treat (mITT) principle in accordance with the randomised intervention. The analysis population will consist of all subjects who complete 12 weeks on treatment and for whom outcomes are available.

Primary outcome analysis will evaluate the impact of treatment allocation (exenatide or placebo) on the difference between MDS UPDRS part 3 OFF medication scores at 96 weeks follow-up adjusting for baseline. The analysis will use a mixed-model approach incorporating information from all follow-up visits that adjusts for baseline Hoehn & Yahr status and the baseline raw value of each outcome measurement. Site will be included as a random effect to account for variability in outcomes between sites, and a random patient/subject effect will accommodate the correlation between repeated outcome measures on the same patient. A significance level of 5% will be used to judge significance for the primary outcome measure.

A planned secondary analysis will compare the difference in MDS-UPDRS part 3 OFF medication scores according to randomisation allocation at 96 weeks, with the scores at 48 weeks. An increase in the advantage at 96 weeks compared to 48 weeks would be evidence that the active drug was slowing down disease deterioration rather than having symptomatic effects only. This could translate to a major population advantage in terms of reduction of morbidity and mortality.

Analyses of the remaining secondary/exploratory outcomes will be undertaken similarly for the difference between groups according to treatment allocation at 48 and 96 weeks follow up adjusting for baseline values of each outcome, and confounding factors such as LED.

For peer review only

Further exploratory analyses will consider whether exenatide can be thought of as disease modifying by comparing slopes between groups at pre-specified periods.

A sensitivity (per protocol) analysis will be performed for the primary outcome measure and will only include those participants who completed the trial in accordance with the approved protocol.

Results on the primary efficacy outcome will be presented by stratum, according to Hoehn & Yahr stage (≤2.0 versus 2.5), and an interaction between Hoehn & Yahr and treatment will be added to the primary analysis model to investigate whether the effect of treatment differs according to the Hoehn & Yahr stage.

All analyses will be performed by the designated Trial Statistician.

Data Management

Data will be entered in the Exenatide-PD3 database by delegated staff at participating sites and members of the Exenatide-PD3 trial team at CCTU. Participants will be given a unique trial PIN (Exnnn). Data will be entered under the PIN onto the central database (InferMed's MACRO stored on the servers based at UCL). The database will be password protected and only accessible to members of the Exenatide-PD3 trial team and external regulators if requested. Video recordings of the MDS-UPDRS will be uploaded onto a secure cloud held by Machine Medicines Technologies (MMT) and used for quality control purposes. Appropriate contractual agreements covering data protection are in place with MMT. All data storage will adhere to GDPR and the Data Protection Act 2018.

An Independent Data Monitoring Committee (IDMC) will be convened including at least 3 individuals independent from the trial team and sponsor who have experience in the conduct of clinical trials for PD. The IDMC will review the trial results and make a recommendation to the Trial Steering Committee (TSC) regarding continuation/ stopping of the trial based on safety data. A statistician independent of the Exenatide-PD3 Trial Team at CCTU will generate summaries of accumulating trial data for the IDMC to review.

UCL is the trial sponsor and has delegated the duties as sponsor to CCTU via a signed letter of delegation. The trial sponsor will take on responsibility for securing the arrangements to initiate, manage and finance the trial. Trial oversight is intended to preserve the integrity of the trial by independently verifying processes and prompting corrective action where necessary. In multi-centre trials this oversight is considered and described both overall and for each recruiting centre by exploring the trial dataset or performing site visits. The Trial Team (TT) will assist with developing the design, co-ordination and day to day operational issues in the management of the trial, including budget management. The Trial Management Group (TMG) will assist with developing the design, co-ordination and strategic management of the trial. The Independent Trial Steering Committee (TSC) is the independent group responsible for oversight of the trial in order to safeguard the interests of trial participants. The TSC will provide advice to the Chief Investigator (CI), CCTU, the funder and sponsor on all aspects of the trial through its independent Chair. The Independent Data Monitoring Committee (IDMC) is the only oversight body that has access to unblinded accumulating comparative data. The IDMC will be responsible for safeguarding the interests of trial participants, monitoring the accumulating data and making recommendations to the TSC on whether the trial should continue as planned. The membership, frequency of meetings, activity (including trial conduct and data review) and authority of each committee will be covered in their respective terms of reference.

Ethics and Dissemination

For peer review only

The trial protocol, all informed consent forms and any material to be given to the prospective participant have received REC (initial date of approval 15/10/2019, REC reference no.19/SC/0447), and other regulatory approvals (EudraCT 2018-003028-35). Further, the trial was registered in clinicatrials.gov NCT004232969 and in ISRCTN (reference 14552789). Subsequent amendments to these documents will be submitted for further approval. The same/amended documents will be submitted for additional local permissions at each clinical site.

This is a Clinical Trial of an Investigational Medicinal Product (IMP) as defined by the EU Directive 2001/20/EC. Therefore, a clinical trial authorisation (CTA) is required in the UK and the trial protocol will therefore be submitted to the UK regulatory authority (MHRA). The progress of the trial, safety issues and reports, including expedited reporting will be reported to the MHRA as required. The protocol, participant information sheet (PIS) and informed consent forms on local headed paper, the REC/HRA and MHRA approvals, schedules of funding and activity (and other trial documentation as needed) have been submitted to the relevant NHS Trust R&D department of each participating site or to other local departments for approval.

Participants will be provided with a PIS and given time to read it fully. Following a discussion with a medical qualified investigator or suitable trained and authorised delegate, any questions will be satisfactorily answered and if the participant is willing to participate, written informed consent will be obtained. During the consent process it will be emphasised that the participant is free to refuse to participate in all or any aspect of the trial, at any time and for any reason, without affecting their treatment. The risk/benefit profile of the trial will be regularly monitored. Consent will be re-sought if new information becomes available that affects the participant's consent in any way.

The rights of the participant to refuse to participate in the trial without giving a reason will be respected and after the participant has entered the trial, the clinician remains free to give alternative treatment to that specified in the protocol, at any stage. The participant remains free to change their mind at any time about the protocol treatment and follow-up without giving a reason and without prejudicing their further treatment. All participants will be made aware of the known adverse reactions.

Discussion

A parallel group design with a washout period has been used previously in the evaluation of potential neuroprotective agents [24,25]. and this was chosen as the design for the previous phase 2 trial. This design is subject to possible long duration symptomatic effects and a lengthy washout period potentially impacts on patient retention and cannot necessarily distinguish a true neuroprotective effect from a symptomatic effect (in view of preservation of healthy behaviours with long term impacts such as exercise) [26]. An alternative approach which we have adopted here is a "Long term simple" design, with longer term follow up to look for a cumulative advantage emerging with prolonged treatment exposure, given the natural history of PD being that of progressive accumulation of motor and non-motor disability [27]. This design helps build on the previous successful clinical trials of exenatide which have introduced a novel, cost effective way of evaluating the potential for disease modifying drugs in PD by recruiting patients already in receipt of conventional dopaminergic treatment, rather than restricting recruitment to incident cases yet to receive dopaminergic treatment. Using this approach, we have successfully demonstrated the potential for rapid recruitment, and improved retention of participants enabling more complete follow up, and a statistically significant advantage in motor scores in people randomised to

For peer review only

exenatide over a 48-week period of treatment exposure. We have considered that an exposure period of 96 weeks would allow exploration of long-term effects of exenatide exposure, while being the maximum period that participants would be willing to accept being allocated placebo. Furthermore, this will provide the opportunity to evaluate whether the 48-week data previously published can be replicated and whether effects at 96 weeks are similar to or greater than those seen at 48 weeks and other earlier timepoints.

Many trials have attempted to evaluate the potential for disease modification using drugs with broad mechanisms of action. The majority have either failed to demonstrate clinical efficacy or provided inconclusive results. Some pertinent reasons for this are a failure of the investigated agent to reach and engage its target and lack of objective measures of true clinical disease progression. Clinical endpoints such as the MDS-UPDRS scale are necessary to ultimately confirm relevance; however, these scales lack sensitivity for capturing disease modification unless very long term follow up data are collected. While our approach of following patients for 2 years in this trial will partially mitigate some of this, the addition and to an extent validation of more detailed approaches through a number of sub studies (imaging, CSF analysis for target engagement and drug levels, and device assisted measurements of real-life motor function) could ultimately provide more holistic and definitive metrics for determining if exenatide does in fact deliver disease modification in PD. This more comprehensive approach to approaching assessments and the consistent signal of benefit noted in two earlier trials provide grounds for optimism that the primary outcome will be achievable. The study opened to recruitment in January 2020 and we expect completion of study analysis by Q3 2024.

Author Contributions:

The study concept and design was conceived by TF. NV and CG will conduct screening and data collection. GA, MC, KM, KC, SH, PL, DA, CC, MH, MS, JD, RH and VL assisted on drafting the protocol. Analysis will be performed by SS, and data entry by AK. NV prepared the first draft of the manuscript. RG served as a patient adviser for the design of the study protocol. All authors provided edits and revised the manuscript for intellectual content.

Acknowledgement

We are grateful to all patients contributing to the design of this study. We thank the Cure Parkinson's Trust and Parkinson's UK for their assistance in the setup and increasing the awareness of this study.

Funding Statement

This work was supported by NIHR grant number 16/167/19. The Cure Parkinson's Trust funded the DATScan Imaging substudy grant number BY-TF021 & the Remote Monitoring substudy grant number MH011.

Competing Interest Statement

For peer review only

None declared

Data Statement

Not applicable at this stage.

Word Count 5729

For peer review only

References:

1 Ray Dorsey E, Elbaz A, Nichols E, *et al.* Global, regional, and national burden of Parkinson's disease, 1990–2016: a systematic analysis for the Global Burden of Disease Study 2016. *Lancet Neurol* 2018;**17**:939–53. doi:10.1016/S1474-4422(18)30295-3

2 Vijiaratnam N, Evans AH. Treatment advances in Parkinson disease: Same script different cast? *J Clin Neurosci* 2018;**47**:354–5. doi:10.1016/j.jocn.2017.10.009

3 Vijiaratnam N, Foltynie T. Therapeutic Strategies to Treat or Prevent Off Episodes in Adults with Parkinson's Disease. *Drugs* 2020;**80**:775–96. doi:10.1007/s40265-020-01310-2

4 Kalia L V., Lang AE. Parkinson's disease. Lancet. 2015;**386**:896–912. doi:10.1016/S0140-6736(14)61393-3

5 Htike ZZ, Zaccardi F, Papamargaritis D, *et al.* Efficacy and safety of glucagon-like peptide-1 receptor agonists in type 2 diabetes: A systematic review and mixed-treatment comparison analysis. *Diabetes, Obes Metab* 2017;**19**:524–36. doi:10.1111/dom.12849

6 Perry T, Lahiri DK, Chen D, *et al.* A Novel Neurotrophic Property of Glucagon-Like Peptide 1: A Promoter of Nerve Growth Factor-Mediated Differentiation in PC12 Cells. *J Pharmacol Exp Ther* 2002;**300**:958–66. doi:10.1124/jpet.300.3.958

7 Perry T, Haughey NJ, Mattson MP, *et al.* Protection and Reversal of Excitotoxic Neuronal Damage by Glucagon-Like Peptide-1 and Exendin-4. *J Pharmacol Exp Ther* 2002;**302**:881–8. doi:10.1124/jpet.102.037481

8 Aviles-Olmos I, Limousin P, Lees A, *et al.* Parkinson's disease, insulin resistance and novel agents of neuroprotection. *Brain* 2013;**136**:374–84. doi:10.1093/brain/aws009

9 Perry T, Lahiri DK, Sambamurti K, *et al.* Glucagon-like peptide-1 decreases endogenous amyloid-β peptide (Aβ) levels and protects hippocampal neurons from death induced by Aβ and iron. *J Neurosci Res* 2003;**72**:603–12. doi:10.1002/jnr.10611

10 Yamamoto H, Lee CE, Marcus JN, *et al.* Glucagon-like peptide-1 receptor stimulation increases blood pressure and heart rate and activates autonomic regulatory neurons. *J Clin Invest* 2002;**110**:43–52. doi:10.1172/JCI15595

11 Drucker DJ, Philippe J, Mojsov S, *et al.* Glucagon-like peptide I stimulates insulin gene expression and increases cyclic AMP levels in a rat islet cell line. *Proc Natl Acad Sci U S A* 1987;**84**:3434–8. doi:10.1073/pnas.84.10.3434

12 Brauer R, Wei L, Ma T, *et al.* Diabetes medications and risk of Parkinson's disease: a cohort study of patients with diabetes. *Brain* 2020;**143**:3067–76. doi:10.1093/brain/awaa262

13 Aviles-Olmos I, Dickson J, Kefalopoulou Z, *et al.* Exenatide and the treatment of patients with Parkinson's disease. *J Clin Invest* 2013;**123**:2730–6.

14 Athauda D, Maclagan K, Skene SS, *et al.* Exenatide once weekly versus placebo in Parkinson's disease: a randomised, double-blind, placebo-controlled trial. *Lancet* 2017;**390**:1664–75. doi:10.1016/S0140-6736(17)31585-4

15 Athauda D, Gulyani S, Karnati HK, *et al.* Utility of Neuronal-Derived Exosomes to Examine Molecular Mechanisms That Affect Motor Function in Patients with Parkinson Disease: A Secondary Analysis of the Exenatide-PD Trial. *JAMA Neurol* 2019;**76**:420–9. doi:10.1001/jamaneurol.2018.4304

16 Crocker JC, Ricci-Cabello I, Parker A, *et al.* Impact of patient and public involvement on

For peer review only

enrolment and retention in clinical trials: Systematic review and meta-analysis. *BMJ* 2018;**363**:k4738. doi:10.1136/bmj.k4738

17 https://www.nihr.ac.uk/documents/improving-inclusion-of-under-served-groups-in-clinical-research-guidance-from-include-project/25435.

18 GIBB WRG, LEES AJ. THE SIGNIFICANCE OF THE LEWY BODY IN THE DIAGNOSIS OF IDIOPATHIC PARKINSON'S DISEASE. *Neuropathol Appl Neurobiol* 1989;**15**:27–44. doi:10.1111/j.1365-2990.1989.tb01147.x

19 Tomlinson CL, Stowe R, Patel S, *et al.* Systematic review of levodopa dose equivalency reporting in Parkinson's disease. *Mov Disord* 2010;**25**:2649–53. doi:10.1002/mds.23429

20 Del Din S, Galna B, Godfrey A, *et al.* Analysis of Free-Living Gait in Older Adults with and Without Parkinson's Disease and with and Without a History of Falls: Identifying Generic and Disease-Specific Characteristics. *Journals Gerontol - Ser A Biol Sci Med Sci* 2019;**74**:500–6. doi:10.1093/gerona/glx254

21 Del Din S, Godfrey A, Galna B, *et al.* Free-living gait characteristics in ageing and Parkinson's disease: impact of environment and ambulatory bout length. *J Neuroeng Rehabil* 2016;**13**:46. doi:10.1186/s12984-016-0154-5

22 Del Din S, Yarnall AJ, Barber TR, *et al.* Continuous Real-World Gait Monitoring in Idiopathic REM Sleep Behavior Disorder. *J Parkinsons Dis* 2020;**10**:283–99. doi:10.3233/JPD-191773

23 Shulman LM, Gruber-Baldini AL, Anderson KE, *et al.* The clinically important difference on the unified parkinson's disease rating scale. *Arch Neurol* 2010;**67**:64–70. doi:10.1001/archneurol.2009.295

24 Datatop: A multicenter controlled clinical trial in early parkinson's disease: Parkinson study group. *Arch Neurol* 1989;**46**:1052–60. doi:10.1001/archneur.1989.00520460028009

25 Olanow CW, Schapira AH, LeWitt PA, *et al.* TCH346 as a neuroprotective drug in Parkinson's disease: a double-blind, randomised, controlled trial. *Lancet Neurol* 2006;**5**:1013–20. doi:10.1016/S1474-4422(06)70602-0

26 Ward CD. Does selegiline delay progression of Parkinson's disease? A critical re-evaluation of the DATATOP study. *J Neurol Neurosurg Psychiatry* 1994;**57**:217–20. doi:10.1136/jnnp.57.2.217

27 Thibault L, Rascol O, Corvol JC, *et al.* New perspectives on study designs for evaluating neuroprotection in Parkinson's disease. *Mov Disord* 2017;**32**:1365–70. doi:10.1002/mds.27055

Figure legend

Figure 1: Outline of Trial design

For peer review only

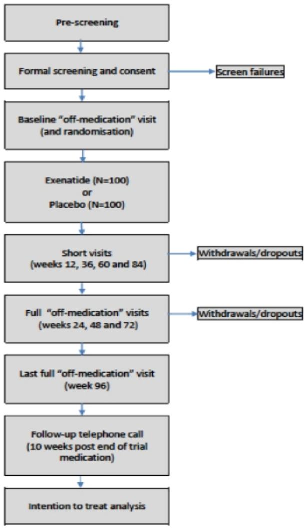

Figure 1: Outline of Trial design

209x296mm (300 x 300 DPI)

Reporting checklist for protocol of a clinical trial.

Based on the SPIRIT guidelines.

Instructions to authors

Complete this checklist by entering the page numbers from your manuscript where readers will find each of the items listed below.

Your article may not currently address all the items on the checklist. Please modify your text to include the missing information. If you are certain that an item does not apply, please write "n/a" and provide a short explanation.

Upload your completed checklist as an extra file when you submit to a journal.

In your methods section, say that you used the SPIRITreporting guidelines, and cite them as:

Chan A-W, Tetzlaff JM, Gøtzsche PC, Altman DG, Mann H, Berlin J, Dickersin K, Hróbjartsson A, Schulz KF, Parulekar WR, Krleža-Jerić K, Laupacis A, Moher D. SPIRIT 2013 Explanation and Elaboration: Guidance for protocols of clinical trials. BMJ. 2013;346:e7586

		Reporting Item	Page Number
**Administrative information**			
Title	#1	Descriptive title identifying the study design, population, interventions, and, if applicable, trial acronym	1
Trial registration	#2a	Trial identifier and registry name. If not yet registered, name of intended registry	2
Trial registration: data set	#2b	All items from the World Health Organization Trial Registration Data Set	2
Protocol version	#3	Date and version identifier	16
Funding	#4	Sources and types of financial, material, and other support	16
Roles and responsibilities: contributorship	#5a	Names, affiliations, and roles of protocol contributors	16

For peer review only

Roles and responsibilities: sponsor contact information	#5b	Name and contact information for the trial sponsor	1
Roles and responsibilities: sponsor and funder	#5c	Role of study sponsor and funders, if any, in study design; collection, management, analysis, and interpretation of data; writing of the report; and the decision to submit the report for publication, including whether they will have ultimate authority over any of these activities	6
Roles and responsibilities: committees	#5d	Composition, roles, and responsibilities of the coordinating centre, steering committee, endpoint adjudication committee, data management team, and other individuals or groups overseeing the trial, if applicable (see Item 21a for data monitoring committee)	6
**Introduction**			
Background and rationale	#6a	Description of research question and justification for undertaking the trial, including summary of relevant studies (published and unpublished) examining benefits and harms for each intervention	3-4
Background and rationale: choice of comparators	#6b	Explanation for choice of comparators	3-4
Objectives	#7	Specific objectives or hypotheses	4
Trial design	#8	Description of trial design including type of trial (eg, parallel group, crossover, factorial, single group), allocation ratio, and framework (eg, superiority, equivalence, non-inferiority, exploratory)	6-7
**Methods: Participants, interventions, and outcomes**			
Study setting	#9	Description of study settings (eg, community clinic, academic hospital) and list of countries where data will be collected. Reference to where list of study sites can be obtained	6-7
Eligibility criteria	#10	Inclusion and exclusion criteria for participants. If applicable, eligibility criteria for study centres and individuals who will	8

For peer review only

		perform the interventions (eg, surgeons, psychotherapists)	
Interventions: description	#11a	Interventions for each group with sufficient detail to allow replication, including how and when they will be administered	12
Interventions: modifications	#11b	Criteria for discontinuing or modifying allocated interventions for a given trial participant (eg, drug dose change in response to harms, participant request, or improving / worsening disease)	12
Interventions: adherance	#11c	Strategies to improve adherence to intervention protocols, and any procedures for monitoring adherence (eg, drug tablet return; laboratory tests)	12
Interventions: concomitant care	#11d	Relevant concomitant care and interventions that are permitted or prohibited during the trial	12
Outcomes	#12	Primary, secondary, and other outcomes, including the specific measurement variable (eg, systolic blood pressure), analysis metric (eg, change from baseline, final value, time to event), method of aggregation (eg, median, proportion), and time point for each outcome. Explanation of the clinical relevance of chosen efficacy and harm outcomes is strongly recommended	9-10
Participant timeline	#13	Time schedule of enrolment, interventions (including any run-ins and washouts), assessments, and visits for participants. A schematic diagram is highly recommended (see Figure)	6-7
Sample size	#14	Estimated number of participants needed to achieve study objectives and how it was determined, including clinical and statistical assumptions supporting any sample size calculations	12
Recruitment	#15	Strategies for achieving adequate participant enrolment to reach target sample size	6
**Methods: Assignment of interventions (for controlled trials)**			
Allocation: sequence generation	#16a	Method of generating the allocation sequence (eg, computer-generated random numbers), and list of any factors for stratification. To reduce predictability of a random sequence, details of any planned restriction (eg, blocking) should be provided in a separate document that is unavailable to those who enrol participants or assign interventions	14

For peer review only

Allocation concealment mechanism	#16b	Mechanism of implementing the allocation sequence (eg, central telephone; sequentially numbered, opaque, sealed envelopes), describing any steps to conceal the sequence until interventions are assigned	14
Allocation: implementation	#16c	Who will generate the allocation sequence, who will enrol participants, and who will assign participants to interventions	14
Blinding (masking)	#17a	Who will be blinded after assignment to interventions (eg, trial participants, care providers, outcome assessors, data analysts), and how	14
Blinding (masking): emergency unblinding	#17b	If blinded, circumstances under which unblinding is permissible, and procedure for revealing a participant's allocated intervention during the trial	14
**Methods: Data collection, management, and analysis**			
Data collection plan	#18a	Plans for assessment and collection of outcome, baseline, and other trial data, including any related processes to promote data quality (eg, duplicate measurements, training of assessors) and a description of study instruments (eg, questionnaires, laboratory tests) along with their reliability and validity, if known. Reference to where data collection forms can be found, if not in the protocol	11
Data collection plan: retention	#18b	Plans to promote participant retention and complete follow-up, including list of any outcome data to be collected for participants who discontinue or deviate from intervention protocols	11
Data management	#19	Plans for data entry, coding, security, and storage, including any related processes to promote data quality (eg, double data entry; range checks for data values). Reference to where details of data management procedures can be found, if not in the protocol	11
Statistics: outcomes	#20a	Statistical methods for analysing primary and secondary outcomes. Reference to where other details of the statistical analysis plan can be found, if not in the protocol	13-14
Statistics: additional analyses	#20b	Methods for any additional analyses (eg, subgroup and adjusted analyses)	13-14

For peer review only

Statistics: analysis population and missing data	#20c	Definition of analysis population relating to protocol non-adherence (eg, as randomised analysis), and any statistical methods to handle missing data (eg, multiple imputation)	13-14
**Methods: Monitoring**			
Data monitoring: formal committee	#21a	Composition of data monitoring committee (DMC); summary of its role and reporting structure; statement of whether it is independent from the sponsor and competing interests; and reference to where further details about its charter can be found, if not in the protocol. Alternatively, an explanation of why a DMC is not needed	7
Data monitoring: interim analysis	#21b	Description of any interim analyses and stopping guidelines, including who will have access to these interim results and make the final decision to terminate the trial	7
Harms	#22	Plans for collecting, assessing, reporting, and managing solicited and spontaneously reported adverse events and other unintended effects of trial interventions or trial conduct	7
Auditing	#23	Frequency and procedures for auditing trial conduct, if any, and whether the process will be independent from investigators and the sponsor	7
**Ethics and dissemination**			
Research ethics approval	#24	Plans for seeking research ethics committee / institutional review board (REC / IRB) approval	14
Protocol amendments	#25	Plans for communicating important protocol modifications (eg, changes to eligibility criteria, outcomes, analyses) to relevant parties (eg, investigators, REC / IRBs, trial participants, trial registries, journals, regulators)	14
Consent or assent	#26a	Who will obtain informed consent or assent from potential trial participants or authorised surrogates, and how (see Item 32)	11-13
Consent or assent: ancillary studies	#26b	Additional consent provisions for collection and use of participant data and biological specimens in ancillary studies, if applicable	11-13
Confidentiality	#27	How personal information about potential and enrolled participants will be collected, shared, and maintained in order to protect confidentiality before, during, and after the trial	11-13

For peer review only

Declaration of interests	#28	Financial and other competing interests for principal investigators for the overall trial and each study site	16
Data access	#29	Statement of who will have access to the final trial dataset, and disclosure of contractual agreements that limit such access for investigators	15
Ancillary and post trial care	#30	Provisions, if any, for ancillary and post-trial care, and for compensation to those who suffer harm from trial participation	15
Dissemination policy: trial results	#31a	Plans for investigators and sponsor to communicate trial results to participants, healthcare professionals, the public, and other relevant groups (eg, via publication, reporting in results databases, or other data sharing arrangements), including any publication restrictions	15
Dissemination policy: authorship	#31b	Authorship eligibility guidelines and any intended use of professional writers	15
Dissemination policy: reproducible research	#31c	Plans, if any, for granting public access to the full protocol, participant-level dataset, and statistical code	15
**Appendices**			
Informed consent materials	#32	Model consent form and other related documentation given to participants and authorised surrogates	11
Biological specimens	#33	Plans for collection, laboratory evaluation, and storage of biological specimens for genetic or molecular analysis in the current trial and for future use in ancillary studies, if applicable	11

The SPIRIT checklist is distributed under the terms of the Creative Commons Attribution License CC-BY-ND 3.0. This checklist was completed on 14. December 2020 using https://www.goodreports.org/, a tool made by the EQUATOR Network in collaboration with Penelope.ai

For peer review only

BMJ Open

Exenatide once weekly over 2 years as a potential disease modifying treatment for Parkinson's disease: protocol for a multi-centre, randomised, double blind, parallel group, placebo controlled, Phase 3 trial, The 'Exenatide-PD3' study

Journal:	*BMJ Open*
Manuscript ID	bmjopen-2020-047993.R1
Article Type:	Protocol
Date Submitted by the Author:	08-Mar-2021
Complete List of Authors:	Vijiaratnam, Nirosen; UCL,<br>Girges, Christine; UCL Queen Square Institute of Neurology, Department of Clinical and Movement Neurosciences; National Hospital for Neurology and Neurosurgery, Department of Clinical and Movement Neurosciences<br>Auld, Grace; University College London<br>Chau, Marisa; University College London, Division of Surgery & Interventional Science<br>Maclagan, Kate; UCL, The Comprehensive Clinical Trials Unit<br>King, Alexa; University College London<br>Skene, Simon; University of Surrey, Surrey Clinical Trials Unit<br>Chowdhury, Kashfia; University College London, Comprehensive Clinical Trials Unit<br>Hibbert, Steve; University College London<br>Morris, Huw ; University College London (UCL)<br>Limousin, Patricia; UCL Queen Square Institute of Neurology, Department of Clinical and Movement Neurosciences; National Hospital for Neurology and Neurosurgery, Department of Clinical and Movement Neurosciences<br>Athauda, Dilan; UCL Queen Square Institute of Neurology, Department of Clinical and Movement Neurosciences; National Hospital for Neurology and Neurosurgery, Department of Clinical and Movement Neurosciences<br>Carroll, Camille; University of Plymouth, Faculty of Medicine and Dentistry<br>Hu, Michele; University of Oxford, Nuffield Department of Clinical Neurosciences<br>Silverdale, Monty; University of Manchester, Department of Neurology and Neurosurgery<br>Duncan, Gordon W; NHS Lothian; University of Edinburgh<br>Chaudhuri, Ray; King\'s College London<br>Lo, Christine; University of Oxford<br>Del Din, Silvia; Newcastle University<br>Yarnall, Alison J; Newcastle University<br>Rochester, Lynn; Newcastle University, Institute of Neuroscience<br>Gibson, Rachel<br>Dickson, John; University College London<br>Hunter, Rachael; University College London, Research Dept of Primary Care and Population Health<br>Libri, Vincenzo; National Hospital for Neurology and Neurosurgery;

	Leonard Wolfson Experimental Neurology Centre<br>Foltynie, Thomas; UCL Queen Square Institute of Neurology, Department of Clinical and Movement Neurosciences; National Hospital for Neurology and Neurosurgery, Department of Clinical and Movement Neurosciences
**Primary Subject Heading**:	Neurology
Secondary Subject Heading:	Medical management, Neurology
Keywords:	Parkinson-s disease < NEUROLOGY, PUBLIC HEALTH, QUALITATIVE RESEARCH

SCHOLARONE™ Manuscripts

I, the Submitting Author has the right to grant and does grant on behalf of all authors of the Work (as defined in the below author licence), an exclusive licence and/or a non-exclusive licence for contributions from authors who are: i) UK Crown employees; ii) where BMJ has agreed a CC-BY licence shall apply, and/or iii) in accordance with the terms applicable for US Federal Government officers or employees acting as part of their official duties; on a worldwide, perpetual, irrevocable, royalty-free basis to BMJ Publishing Group Ltd ("BMJ") its licensees and where the relevant Journal is co-owned by BMJ to the co-owners of the Journal, to publish the Work in this journal and any other BMJ products and to exploit all rights, as set out in our licence.

The Submitting Author accepts and understands that any supply made under these terms is made by BMJ to the Submitting Author unless you are acting as an employee on behalf of your employer or a postgraduate student of an affiliated institution which is paying any applicable article publishing charge ("APC") for Open Access articles. Where the Submitting Author wishes to make the Work available on an Open Access basis (and intends to pay the relevant APC), the terms of reuse of such Open Access shall be governed by a Creative Commons licence – details of these licences and which Creative Commons licence will apply to this Work are set out in our licence referred to above.

Other than as permitted in any relevant BMJ Author's Self Archiving Policies, I confirm this Work has not been accepted for publication elsewhere, is not being considered for publication elsewhere and does not duplicate material already published. I confirm all authors consent to publication of this Work and authorise the granting of this licence.

For peer review only

Exenatide once weekly over 2 years as a potential disease modifying treatment for Parkinson's disease: protocol for a multi-centre, randomised, double blind, parallel group, placebo controlled, Phase 3 trial, The 'Exenatide-PD3' study

Nirosen Vijiaratnam[1,2], Christine Girges[1,2], Grace Auld[3], Marisa Chau[3], Kate MacLagan[3], Alexa King[3], Simon Skene[4], Kashfia Chowdhury[3], Steve Hibbert[3], Huw Morris[1,2] Patricia Limousin[1,2], Dilan Athauda[1,2], Camille B. Carroll[5,6], Michele T. Hu[7-9], Monty Silverdale[10], Gordon W Duncan[16,17] Ray Chaudhuri[14], Christine Lo[8], Silvia Del Din[15], Alison J Yarnall[15,18], Lynn Rochester[15], Rachel Gibson, John Dickson[11], Rachael Hunter[12], Vincenzo Libri[2,13] & Thomas Foltynie[1,2]

1. Department of Clinical and Movement Neurosciences, UCL Queen Square Institute of Neurology, London, UK.

2. The National Hospital for Neurology and Neurosurgery, Queen Square, London, UK.

3. Comprehensive Clinical Trials Unit, University College London, London, UK

4. Department of Clinical and Experimental Medicine, University of Surrey, Guildford, UK.

5. Applied Parkinson's Research Group, University of Plymouth, Faculty of Health, Plymouth, Devon, United Kingdom.

6. University Hospitals Plymouth NHS Trust, Plymouth, Devon, United Kingdom.

7. Nuffield Department of Clinical Neurosciences, Division of Clinical Neurology, University of Oxford, Oxford, UK.

8. Oxford Parkinson's Disease Centre, University of Oxford, Oxford, UK.

9. Department of Clinical Neurology, Oxford University Hospitals NHS Foundation Trust, Oxford, UK.

10. Department of Neurology and Neurosurgery, Salford Royal NHS Foundation Trust, Manchester Academic Health Science Centre, University of Manchester, Greater Manchester, UK.

11. Institute of Nuclear Medicine, University College London, London, UK.

12. Research Department of Primary Care and Population Health, Royal Free Medical School, University College London, Royal Free Campus, Rowland Hill Street, London, NW3 2PF, United Kingdom.

13. National Institute for Health Research, University College London Hospitals NHS Foundation Trust, Clinical Research Facility and Leonard Wolfson Experimental Neurology Centre.

14. Parkinson Foundation International Centre of Excellence, King's College Hospital and King's College London

15. Translational and Clinical Research Institute, Faculty of Medical Sciences, Newcastle University, Newcastle upon Tyne

16. Western General Hospital, NHS Lothian, Edinburgh, UK

17. University of Edinburgh, Edinburgh, UK

18. Newcastle upon Tyne NHS Foundation Trust, Newcastle, UK

For peer review only

Corresponding author:

Prof Thomas Foltynie

Department of Clinical and Movement Neurosciences,

UCL Queen Square Institute of Neurology,

London, UK.

Email: t.foltynie@ucl.ac.uk

Trial Registration Number: Clinicaltrials.gov identifier: NCT04232969, ISRCTN14552789

Key Words:

Exenatide; Parkinson's disease; randomised control trial.

Abstract

Introduction

Parkinson's disease (PD) is a common neurodegenerative disorder with substantial morbidity. No disease modifying treatments currently exist. The glucagon like peptide-1 receptor agonist exenatide has been associated in single centre studies with reduced motor deterioration over 1 year. The aim of this multicentre UK trial is to confirm whether these previous positive results are maintained in a larger number of participants over two years and if effects accumulate with prolonged drug exposure.

Methods and analysis

This is a phase 3, multi-center, double-blind, randomised, placebo-controlled trial of exenatide at a dose of 2 mg weekly in 200 participants with mild to moderate PD. Treatment duration is 96 weeks. Randomisation is 1:1, drug to placebo. Assessments are performed at baseline, week 12, 24, 36, 48, 60, 72, 84, and 96 weeks.

The primary outcome is the comparison of MDS-UPDRS part 3 motor sub-score in the practically defined OFF medication state at 96 weeks between participants according to treatment allocation. Secondary outcomes will compare the change between groups among other motor, non-motor and cognitive scores. The primary outcome will be reported using descriptive statistics and comparisons between treatment groups using a mixed model, adjusting for baseline scores. Secondary outcomes will be summarised between treatment groups using summary statistics and appropriate statistical tests to assess for significant differences.

Ethics and dissemination

This trial has been approved by the South Central-Berkshire Research Ethics Committee and the Health Research Authority. Results will be disseminated in peer-reviewed journals, presented at scientific meetings and to patients in lay-summary format.

Article Summary

Strengths and Limitations of this Study

For peer review only

- This is the protocol for the first phase 3 double-blind, randomised, placebo controlled trial of exenatide in Parkinson’s disease (PD).
- This study uses novel secondary outcome measures in sub-studies (cerebrospinal fluid analysis, dopamine transporter imaging, and digital technology measurement devices) which should provide a more sensitive and comprehensive assessment of potential disease modification.
- Although the 2 year follow-up period should provide a more definitive signal on disease modification this will take longer to report findings and has risks for long-term patient retention

Introduction

Parkinson’s disease (PD) is the second most common neurodegenerative disease affecting over 10 million people worldwide and its prevalence is increasing [1]. Symptomatic treatments are available and mainly focus on dopamine replacement strategies [2,3]. Such therapies provide improvements in the core motor features of PD: tremor, limb rigidity and slowness of movement (bradykinesia) [4]. These symptomatic treatments do not impact on the progressive nature of the disease nor the majority of the non-motor symptoms (NMS). Moreover, with time, some patients will develop dopamine-refractory gait and balance problems leading to falls and risk of fractures; speech and swallowing problems leading to difficulty in communication and aspiration pneumonia;, cognitive impairment, visual hallucinations and dementia with mounting care needs.[3,4]. These complications result in increased dependence, care giver strain, need for 24 hour care and death. Therefore, Parkinson's disease is a growing problem for individuals, healthcare and society making the development of disease modifying treatments imperative.

Exenatide (Exendin-4) is a licensed and effective treatment for patients with type 2 diabetes mellitus (T2DM) [5]. It is an agonist for the Glucagon-like peptide 1 (GLP-1) receptor and in the presence of elevated blood glucose stimulates insulin release. It also increases pancreatic beta islet cell mass and reduces apoptosis. Exenatide has been the subject of multiple phase 3 trials in patients with type 2 diabetes and was granted a license for the treatment of type 2 diabetes in 2006 [5].

In parallel with the confirmation of the beneficial effects of exenatide on glucose control, laboratory work has showed that exenatide has beneficial effects on neurons in vitro [6]. Exenatide induces neurite outgrowth, promotes neuronal differentiation and rescues degenerating neuronal cells while also reversing neurotoxin induced damage in animal models [6,7]. These neurotrophic properties have sparked interest regarding its potential use as a neurodegenerative disease modifying agent [8,9].

The specific relevance of exenatide to PD has also been extensively evaluated. Exenatide has been shown to increase transcription of tyrosine hydroxylase (TH) (the rate limiting enzyme in dopamine synthesis) in brainstem catecholaminergic neurons [10]. Furthermore, stimulation of GLP-1 receptors may have beneficial effects on the neurodegenerative processes of PD through downstream cellular pathways [6,11]. These findings are further supported by a recent study suggesting a reduced future risk of developing PD in T2DM patients treated with GLP-1 agents [12].

To investigate the potential effects of exenatide in patients with PD, an investigator-initiated pilot trial was undertaken [13]. This open label, parallel group, randomised controlled trial evaluated the tolerability of exenatide (Byetta 10mcg twice daily) in 45 patients with moderately severe PD (Hoehn and Yahr stage of less than 2.5) over an exposure period of 48 weeks with a subsequent washout

For peer review only

period of 12 weeks. This showed an advantage of 4.9 points in in the Movement Disorders Society Unified Parkinson's Disease Rating Scale (MDS-UPDRS) part 3 (motor sub-score) in exenatide treated patients at 12 months which persisted even after a 12 week washout period. Clinically important differences in cognition were also noted. Serial DaTscan imaging showed no progression between baseline and 48 weeks in the exenatide treated patients [13].

A further Phase 2 double blind randomised controlled trial evaluating the effects of exenatide in 60 patients with PD has subsequently been performed [14]. Patients were randomised to self-injection of a long acting form of exenatide, (Bydureon 2mg) once weekly, or matched placebo for 48 weeks. Detailed assessments every 12 weeks for the duration of the treatment and a further assessment at the 60 week time point to explore any lasting effects following washout of the trial medication were performed. Patients receiving exenatide had a mean 3.5 point advantage in their MDS-UPDRS part 3 OFF medication scores compared to patients receiving placebo at the 60 week timepoint. Biological specimens collected from trial participants confirmed changes according to treatment with exenatide in downstream cellular effector pathways [15].

The current trial objective (Box 1) is to confirm or refute whether the previous positive results can be reproduced in a multicentre trial design, including a larger number of participants evaluated over twice as long a period as previously. An important secondary objective is to explore if positive effects seen after 48 weeks of exenatide exposure remain static or increase in amplitude by the 96-week time point. The hypothesis is that exenatide will be associated with reduced MDS-UPDRS part 3 scores at the 96-week time-point. The overriding priority for this trial is to provide evidence to support or refute any signal of efficacy of exenatide in PD, and thus provide the justification for rapid further investment in this drug if appropriate. In parallel with this, is the aim to explore whether any biological effect(s) of exenatide, relevant to PD, are purely symptomatic effects as opposed to disease modifying effects.

For peer review only

Box 1 Trial Objectives

Primary

- Compare the effectiveness of exenatide once weekly versus placebo on the MDS-UPDRS part 3 motor sub-score in the “practically defined OFF medication state” in patients with PD (Change in the MDS-UPDRS part 3 score reflects accumulation of motor deficit and therefore is a measure of PD motor progression)

Secondary

- Compare differences at 48 and 96 weeks between the exenatide and placebo trial arms in:
  - MDS-UPDRS part 1, 2, 3 and 4 ON medication scores
  - Timed Walk assessment ON and OFF medication
  - Montreal Cognitive Assessment (MoCA)
  - Safety and tolerability of exenatide as indicated by changes in vital signs, weight, clinical laboratory measures and adverse events
  - Patient Health Questionnaire (PHQ-9)
  - Unified Dyskinesia Rating Scale (UDysRS)
  - Parkinson’s Disease 39 item Quality of Life questionnaire (PDQ-39)
  - Levodopa equivalent dose change
  - 3 day Hauser diary of PD state (Time-On, Off, Non troublesome Dyskinesia, Troublesome dyskinesia, Asleep)

- Compare differences in total values over 96 weeks between the exenatide and placebo trial arms in:
  - Health and social care resource use on the modified Client Service Receipt Inventory (CSRI)
  - Health and social care costs
  - Paid and unpaid carer costs
  - Quality adjusted life years (QALYs) calculated using the EQ-5D-5L tariff adjusting for baseline.

- Compare differences between scores at 48 and 96 weeks between the exenatide and placebo trial arms in:

  - MDS-UPDRS part 3 Motor subsection OFF medication score

Exploratory

Compare differences between slopes at pre-specified periods between exenatide and placebo trial arms for key outcomes to investigate whether exenatide can be considered disease modifying.

Methods

This trial protocol was designed using the University College London (UCL) Comprehensive Clinical Trials Unit (CCTU) Protocol template. The trial is sponsored by UCL and coordinated by the CCTU. The protocol was designed to provide information about procedures for entering participants into the trial, and sufficient detail to enable: an understanding of the background, rationale, objectives, trial population, intervention, methods, statistical analyses, ethical considerations, dissemination plans and administration of the trial; replication of key aspects of trial methods and conduct; and appraisal of the trial's scientific and ethical rigour from the time of ethics approval through to dissemination of the results**.** All stake holders (research team, sponsor, CCTU and oversight committees) were involved in the design and approval of the protocol. A particular emphasis was given to patient input in the trial design. This patient and public involvement (PPI) approach has proven to be of value in other studies [16] and was harnessed to improve the overall study design. A focus group meeting with patients was organized in the protocol design stages to obtain feedback from patients which led to a number of amendments prior to submission, including the maximum overall trial duration of 96 weeks, and the use of OFF-medication assessments. Two PPI representatives will serve on the trial steering committee (TSC) and will continue to provide regular input throughout recruitment. Patients will also be provided access to the trial website and a link to the protocol and patient information sheets (PIS) on request and will be given the opportunity to continue to provide comments and contact researchers to further discuss their input. The INCLUDE guidance [17] is an NIHR led initiative to improve inclusion of under-served groups. The design of the trial is mindful of the value of the steps outlined in this initiative and aims to incorporate its recommendations into overall trial recruitment with the overarching aim of providing better access and quality care to under-served patient groups.

Patient and Public Involvement:

In the development of this protocol, a formal meeting hosted by the Cure Parkinson's Trust was held with 6 patients with PD to obtain patient feedback on the overall trial design and logistical aspects of the trial that could potentially impede recruitment and retention. The aims and objectives of the trial were discussed including the importance of distinguishing between symptomatic and disease modifying effects of exenatide. Patient feedback was clear that a 2 year period would be the maximal acceptable duration of self-administration of placebo, therefore the trial duration was reduced from the original planned 3 year duration to 96 weeks. The use of weekly self-administered injections, and attendance in the off-medication state to assess PD severity was discussed in detail and considered acceptable. The recruitment strategy has used the patient networks of the Cure Parkinson's Trust and Parkinson's UK to increase the awareness of the trial. Patients and patient representatives are included in the Trial Steering committee. At the end of the study, all participants will be notified of their randomisation allocation and of the main study results. The results will be presented at meetings convened for patient groups and published in open access peer reviewed publications.

Trial design

This is a simple parallel group multicentre phase 3, double-blind, randomised, placebo-controlled trial which includes a 96-week exposure period. Detailed evaluations of all participants will take place at screening, baseline, 24, 48, 72 and 96 weeks (Figure 1). Participants will also attend on a 12 weekly basis to collect supplies of Investigational Medicinal Product (IMP). Participants will be randomly allocated to receive either exenatide extended release 2mg subcutaneous injection (Bydureon) once weekly for 96 weeks n=100, or exenatide extended release placebo subcutaneous injection once weekly for 96 weeks n=100. In addition, participants will be randomised using a minimisation algorithm (with a random element incorporated) balancing by research site, participants with greater (Hoehn & Yahr stage 2.5) or lesser (Hoehn & Yahr stage 2.0 or less) PD severity (in the ON medication state), and participation in the sub-studies (Remote Monitoring, Imaging, or not participating).

Participants & recruitment

Patients are eligible for screening if they have a clinical diagnosis of PD. The Queen Square brain bank criteria[18] can be also be used to validate the diagnosis and ensure consistency of diagnosis between sites, however, this is not a formal inclusion criterion. The relevance of a positive family history of PD, or a confirmed genetic basis for an individual's symptoms will be evaluated in the context of other clinical features in determining diagnosis and eligibility. Key inclusion and exclusion criteria are summarised in Box 2.

In a post hoc analysis of the Exenatide PD phase 2 trial, younger patients with shorter disease duration had the best outcomes [19]. While aware of this, we feel it is important to collect evidence to determine whether exenatide has beneficial effects on cognition and axial features of PD, and thus took the decision to keep the inclusion criteria broad to improve our chances of detecting effects on these other outcomes and also ensuring that the results will be applicable to the broadest population of PD patients

Participants will typically be recruited through specialist movement disorders clinics at trial sites. The trial will be advertised online by the Parkinson's UK website, the Cure Parkinson's Trust and the NIHR Clinical Research Network websites and will be registered on ClinicalTrials.gov and the ISRCTN registry. Trial advertisements will direct participants to contact teams in order to be provided with a patient information sheet and a reply slip to confirm ongoing interest and to organise a pre-screening telephone call to discuss eligibility and suitability for the study. It is anticipated that recruitment will be completed from six UK sites (National Hospital for Neurology and Neurosurgery (Queen Square, London), King's College Hospital NHS Foundation Trust (London), Oxford University Hospitals NHS Foundation Trust (Oxford), Derriford University Hospital (Plymouth), Salford Royal Hospital (Manchester) and Western General Hospital & Royal Infirmary of Edinburgh (Edinburgh). Recruitment rates will be carefully monitored throughout the trial to inform on the total number of sites required to ensure final recruitment milestones will be reached. All patient assessments will be performed at hospitals in the UK, after a site initiation visit has been performed. The trial began recruitment on January 20th 2020 and will aim to complete all assessments by 30th September 2023.

For peer review only

Box 2 Key inclusion and exclusion criteria for study

Key inclusion criteria

- Diagnosis of Parkinson's disease PD based on review of the participant's clinical history, examination findings and response to PD medications. The Queen Square brain bank criteria [18] can be also be used to assist the diagnosis, however this is not a formal inclusion criterion. The relevance of a positive family history of PD, or a confirmed genetic basis for an individual's symptoms will be evaluated in the context of other clinical features in determining diagnosis and eligibility.
- Hoehn and Yahr stage ≤2.5 in the ON medication state. This implies that all patients will be mobile without assistance during their best "ON" medication periods.
- Between 25 and 80 years of age.
- On dopaminergic treatment for at least 4 weeks before enrolment. All participants must have had previous or ongoing exposure to dopaminergic treatment either as L-dopa or a dopamine agonist. If L-dopa has been stopped due to side effects or lack of response, the local PI should further confirm that the participant has clinical symptoms and signs and/or radiological investigations consistent with a diagnosis of Parkinson's disease.
- Ability to self-administer, or to arrange carer administration of trial medication.
- Documented informed consent to participate.

Key exclusion criteria

- Diagnosis or suspicion of other cause for Parkinsonism. Patients with clinical features indicating a diagnosis of Progressive Supranuclear Palsy, Multiple Systems Atrophy, Drug induced Parkinsonism, Dystonic tremor or Essential tremor will not be recruited.
- Patients unable to attend the clinic visits in the practically defined OFF medication state.
- Body mass index <18.5. (Exenatide is known to cause weight loss therefore individuals that may not tolerate further weight loss will not be recruited).
- Known abnormality on CT or MRI brain imaging considered likely to compromise compliance with trial protocol.
- Significant cognitive impairment defined by a score <21 on the Montreal Cognitive Assessment.
- Concurrent severe depression defined by a score ≥16 on the Patient Health Questionnaire (PHQ-9)
- Prior intra-cerebral surgical intervention for Parkinson's disease. Patients who have previously undergone Deep Brain Stimulation, intra-cerebral administration of growth factors, gene therapy or cell therapies will not be eligible.
- Previous participation in one of the following Parkinson's disease trials (Biogen SPARK trial, Prothena Pasadena trial, Sanofi Genzyme MOVES-PD trial, UDCA-PD UP Study or any other trial still considered to involve a potentially PD modifying agent). In the event of any uncertainty, the Chief Investigator will discuss the relevance of exposure to any other specific trials/experimental agents with the local Principal Investigator before recruitment eligibility is confirmed.
- Participation in another clinical trial of a device, drug or surgical treatment within the last 30 days.
- Previous exposure to exenatide.
- Impaired renal function with creatinine clearance <50ml/min.
- History of pancreatitis. Screening serum amylase value must fall within laboratory normal range +/- 50%.
- Type 1 or Type 2 Diabetes mellitus.
- Severe gastrointestinal disease (e.g. gastroparesis)
- Hyperlipidaemia. A lipid profile will be tested at the screening visit. Cholesterol or Triglyceride levels greater than 2 x the upper limit of normal will raise suspicion of a familial or acquired hyperlipidaemia and will prompt referral to a relevant specialist for investigation and treatment.
- History or family history of medullary thyroid cancer (MTC). Undiagnosed neck lump, hoarse voice or difficulty swallowing (not attributable to PD diagnosis).
- Multiple endocrine neoplasia 2 (MEN2) syndrome.
- Hypersensitivity to any of exenatide's excipients.
- Females that are pregnant or breast feeding. There are no safety data regarding exenatide use in pregnancy.
- Women of child bearing potential who are unwilling or unable to use an acceptable method to avoid pregnancy for the entire trial period and up to 3 months after the last dose of trial medication. Female participants who are able to become pregnant (defined as women of child-bearing potential) will undergo a pregnancy test prior to randomisation and will be asked at each visit to confirm regular use of an effective method of contraception
- Participants who lack the capacity to give informed consent.
- Any medical or psychiatric condition or previous conventional/experimental treatment which in the investigator's opinion compromises the potential participant's ability to participate.

Outcomes

Safety monitoring

Safety and tolerability of exenatide as indicated by changes in vital signs, weight, clinical laboratory measures and adverse events (AEs) will be recorded and monitored throughout. Each patient will have their pulse, blood pressure and weight documented at screening and at each follow up visit. Exenatide is known to cause weight loss. Participants' height will be recorded at screening to enable calculation of body mass index. At each visit, participants are asked to report any AEs that have occurred since the previous visit. AEs may also be detected by the study team reviewing the patient or through notification by the participant's primary care physician. All AEs will be assessed by a study doctor for their severity, likely relationship to study drug and required action by a study doctor not involved in the blinded assessment of the patient. All SAEs will be recorded and reported to the sponsor regardless of relation to trial treatment. Any suspected unexpected serious adverse reactions will be reported to the sponsor immediately to allow facilitation of unblinding as necessary. All AEs reported will be reviewed by the trial management group, trial steering group and monitored by an independent data monitoring committee. Unblinding requests from other clinicians responsible for a patient's care will be handled by the principal investigator (PI) at each site. The PI at each site may also choose to unblind a participant in response to reported AEs as they are reported, if judged to be clinically necessary.

Primary Outcome

The ***MDS-UPDRS part 3 motor*** OFF medication score is a widely accepted measure of the motor disability of PD. The scale is performed in the ON medication state and in the practically defined OFF medication state. This is defined as the score obtained in a patient who has withheld all short acting conventional PD medications for at least 8 hours and all long acting conventional PD medications for at least 36 hours. Comparison of MDS-UPDRS part 3 motor sub-score in the practically defined OFF medication state at 96 weeks between participants according to treatment allocation and adjusted for baseline will be the primary outcome. The scores for these assessments will be collected and recorded by trained clinical trial personnel (if possible, the same person will rate these assessments at each site to minimise inter-rater variability). With consent, these assessments will be video recorded as part of a MDS-UPDRS automated scoring sub study though the availability of these videos will also enable repeated independent scoring to be performed if there are concerns raised about data quality from a specific site/rater.

Secondary Outcomes

Comparisons at 48 and 96 weeks between participants according to treatment allocation will also be performed for each of the secondary outcomes listed below.

The ***MDS-UPDRS part 3 motor score in the practically defined OFF medication state*** at 48 weeks. Whereas the analysis of the 96-week scores according to randomisation group will represent the primary outcome for this trial, differences emerging at 48 weeks and also the difference between scores at 48 weeks and 96 weeks will be important secondary outcomes.

MDS-UPDRS part 1, 2, 3 and 4 ON medication scores. Part 3 of the MDS-UPDRS as well as the other elements (Part 1,2 & 4) of the scale will also be evaluated in the presence of conventional PD medication (ON state) to evaluate any change in some of the non-motor symptoms of PD, activities of daily living and the complications of chronic PD treatment.

Montreal Cognitive assessment (MoCA). This scale is a validated global measure of cognitive ability. This will be assessed in the ON medication state.

Timed Tests. Participants will be asked to perform a ***Sit-stand-walk timed test*** in both the OFF medication and ON medication state. The timed Sit-stand-walk test will incorporate time taken from seated position to stand and walk 10 metres, turn and return to original seated position.

Unified dyskinesia rating scale (UDysRS). This is considered to be the most useful and objective way of quantifying dyskinesia severity. This will be assessed in the ON medication state.

Patient Health Questionnaire-9 (PHQ-9). This scale allows for self-quantification of depression severity. This will be assessed in the ON medication state.

Non-motor symptom scale (NMSS). This validated scale is a tool to collect data on the frequency and severity of 30 non-motor symptoms sometimes experienced by PD patients. This will be assessed in the ON medication state.

The Parkinson's Disease Questionnaire (PDQ-39). This is the standard disease specific measure of quality of life in PD comprising 39 questions. It has been extensively validated in previous studies.

Levodopa equivalent dose (LED). To facilitate comparisons between patients taking different regimes of conventional PD medications, a set of conversion factors have been used to convert each of the commonly used PD medications to an LED of each of their medications can then be summed for inter-patient / inter-group comparisons.[20]

EQ-5D-5L. This is a simple, 5 question form and visual analogue scale that allows calculation of quality adjusted life years (QALY) to enable health economic analyses to be performed.

The Client Service Receipt Inventory (CSRI). Health and social care resource use. Self-completed health care, social care and paid/unpaid carer resource use questionnaire asking about primary and secondary care resource use relevant to Parkinson's and impact on carers in the past 6 months.

3-day Hauser diary of PD state (Time- On, Off, Troublesome Dyskinesia, Non-troublesome dyskinesia, Asleep). Diary data allows quantification of the amount of time during a 3-day period that patients spend in the varying states of movement ability.

Ancillary Studies

There are four optional sub-studies linked to the main trial:

1. Genetics sub-study: To try to identify genetic markers that may be associated with subtypes of PD or variation in treatment responsiveness.

2. Cerebrospinal Fluid (CSF) sub-study: To determine whether any CSF changes associated with PD are influenced by exposure to exenatide. These may include alpha synuclein monomers or oligomers, neuroinflammatory markers, and exosomal contents.

3. Remote Monitoring of PD Symptoms sub-study: To help determine whether measurement of PD symptoms using digital technology may be a more sensitive measure of change with active drug versus placebo compared to the MDS UPDRS 3 in the OFF and ON medication states. This will form two separate measurements comprising 1) home-based smartphone and 2) real-world gait/walking activity monitoring.[21],[22],[23] This aims to generate precision data, providing person-specific distributions of outcomes and may be able to better delineate baseline clinical features.

For peer review only

4. DaTSCAN (Imaging sub-study): To determine if change in dopamine transporter availability in the caudate and putaminal nuclei as measured by quantitative DaTSCAN signal is influenced by exposure to exenatide compared to placebo.

Visits

The overall progression of assessments are summarised in figure 1. While we expect to undertake all assessments in respective clinical units, provision has been made in line with INCLUDE guidance for the possibility of home visit assessments to be performed when patient specific situations (e.g. inability to travel due to coronavirus restrictions, worsening 'OFF' state over progression of trial) necessitate this. We hope that this provision will aid overall trial retention while enhancing recruitment of patients from typically less well represented demographics (e.g. rural geographic regions, patients lacking private travel facilities).

Screening Visit

Written informed consent to enter and be randomised into the trial will be obtained from participants, after explanation of the aims, methods, benefits and potential hazards of the trial and before any trial-specific procedures are performed or any blood is taken for the trial. Patients will be screened using the history of their Parkinson's disease, supported by any available clinical correspondence according to usual standard of care.

The collection of the following scales will evaluate patient eligibility: MoCA, PHQ-9, as well as blood tests (full blood count, urea & electrolytes, creatinine, liver function tests, HbA1C, C-peptide, coagulation, serum amylase, thyroid function tests, blood glucose, insulin and lipid profile, and a pregnancy test for women of child bearing potential). Tests can be repeated between screening and baseline visits, if required to confirm eligibility. Abnormalities detected that warrant further management e.g. newly diagnosed diabetes will be referred for appropriate medical evaluation.

Patients recruited to the DATSCAN substudy will have imaging performing prior to their baseline visit.

Baseline visit and randomisation

Previously defined primary and secondary outcome measures will be performed in the 'ON' and 'OFF' states as outlined below. Patients' LED will be noted. Randomisation to either exenatide or placebo will be administered using a centralised, web-based system (www.sealedenvelope.com). All assessments related to sub-studies will also be performed prior to trial medication administration.

Assessment Procedures

After the Screening Visit, the named site clinical staff member will call the participant to remind them of the need to stop taking their regular PD medication prior to their next trial visit and to attend in a fasted state (prior to visits 2, 4, 6, 8, 10). The MDS-UPDRS part 3 and Timed Walk assessments will be initially performed in the OFF state. This assessment in both the 'OFF' and 'ON' states will be performed with video recording to facilitate the possibility of a re-review if necessary. Remote monitoring assessments will be conducted at this point at selected sites in patients consenting to participate in this sub-study. While waiting for medications to work, participants will self-complete the MDS-UPDRS parts 1, 2 and 4, PDQ-39, EQ-5D-5L and CSRI. The MDS-UPDRS part 3 and Timed Walk assessments will be repeated 1 hour after the participant has taken their routine medications - the ON medication state. After completion of the MDS-UPDRS and Timed Walk

For peer review only

assessments in the ON medication state, each participant will be assessed using the MoCA, NMS scale, UDysRS and PHQ-9. This will occur in alternate post-randomisation assessments (at visits 2, 4, 6, 8, 10). At selected centres participants in the CSF sub-study will have a CSF sample taken via lumbar puncture. Ten weeks after the last trial medication administration, a staff member will call the participant to collect details of any adverse events that have occurred after the participant stops taking the trial medication. Participants will complete the 3 Day Hauser Diary prior to visits 2, 6 and 10 and return the diary back to the research team at the respective study visits. At each of the visits 2, 4, 6, 8, and 10, a blood sample will be collected and processed for storage for future analysis.

The DaTSCAN imaging sub-study will be performed at the UCLH site on all consenting sub-study participants; scans will be performed prior to visit 2 and after visit 10.

The option for performing a remote assessment will be provided to patients for safety monitoring visits in view of the coronavirus pandemic.

For peer review only

Intervention

Each dose of exenatide 2 mg (powder and solvent for prolonged release, suspension for injection, prefilled pen) is supplied as a single use injection pen for subcutaneous administration by the patient on a weekly basis. The placebo (inactive powder and solvent for prolonged release, suspension for injection, prefilled pen) is supplied as an identical injection pen for subcutaneous administration by the patient on a weekly basis. The trial medication will be refrigerated and stored at 2-8°C. Both exenatide and placebo will be supplied by AstraZeneca as unlabelled prefilled pens in bulk and in accordance with Good Manufacturing Practice (GMP). Labelling, packaging and release of packed trial medications will be managed by the Sponsor’s contracted company following GMP. The labels will be prepared in accordance with GMP Annex 13 (Supplementary Materials 1) requirements for labelling and local regulatory guidelines. The trial medications will be released ahead of trial use.

Site trial staff will be trained on the use of exenatide using an online teaching video, accompanying product literature and the Investigator’s Brochure (IB). Patients will be taught how to perform the subcutaneous injections by the clinical trial team using the online video, demonstration packs and written literature. They will be told about common adverse reactions previously reported e.g. nausea, vomiting, diarrhoea and weight loss by the clinical trial team, and will be advised on the processes for safety reporting. In the event that exenatide injections will be administered by care-givers (e.g. spouse), their willingness to perform this will be documented and they will be trained using the online teaching video. It will also be ascertained that the care-giver either lives with the PD patient or confirms their willingness to meet with the PD participant on a weekly basis to administer the injections for the 96 week period of the trial.

Patients who meet eligibility criteria at the screening visit will be randomly assigned to receive 96 weeks of double-blind treatment with either exenatide or placebo (2mg once weekly) in a 1:1 ratio. The first dose will be administered by the patient in clinic following injection training and subsequent injections will be at home. Injections will be self-administered by the participants, or administered by their carer, into the participants’ abdomen, arm, thigh or buttocks every 7 days. Participants will be provided with a link to the injection pen training video and a REC approved injection administration training sheet.

Sample Size

The sample size is based on the detectable effect size (primary outcome is the MDS-UPDRS motor subsection in the OFF medication state) for a two-arm (exenatide versus placebo) parallel-group trial design. The calculations assume a common standard deviation of 13.5, and a correlation of 0.70 between baseline and follow up MDS-UPDRS measurements. These estimates are reasonable based on data from the previous exenatide-PD trial[14]. On this basis, 160 evaluable participants divided equally between the 2 groups is sufficient to detect a difference of 5.0 MDS-UPDRS part 3 points in the OFF medication state between the 2 groups adjusting for baseline MDS-UPDRS part 3 OFF scores, with 90% power and at a significance level of 0.05. Assuming 20% attrition (withdrawal/loss to follow up), 200 participants will be recruited. Participants who withdraw from the trial will not be replaced. Participants who withdraw from trial treatment should remain in the trial for the purpose of follow-up and data analysis. This effect size is a reasonable expectation based on the previously collected pilot data and would represent a clear demonstration of the efficacy of exenatide on the motor severity of PD.

It is also anticipated that the difference in scores in the ON medication state will be greater at 96 weeks than at the earlier time points. The expected rate of change in PD severity in the first 5 years after PD diagnosis in the ON medication state is 1 MDS-UPDRS part 3 point per year. A predicted advantage of 2 points in ON scores over 96 weeks would thus equate to an advantage in the rate of disease progression above and beyond that achievable with conventional dopaminergic medication and would be a further clear signal that continued use of exenatide is consistent with not only long-term disease modifying effects, but even demonstration of a small change of 2.5 points in the MDS-UPDRS motor score would constitute a clinically important difference[24] and potentially an advantage in day to day functional impairment and overall improvement in quality of life in the short term.

Statistical Analysis

A full statistical analysis plan (SAP) will be written and approved by the Trial Steering Committee prior to database lock. All analyses will be undertaken according to a modified intention-to-treat (mITT) principle in accordance with the randomised intervention. The threshold for the analysis population will be participants who complete 12 weeks on treatment and for whom outcomes are available.

Primary outcome analysis will evaluate the impact of treatment allocation (exenatide or placebo) on the difference between MDS UPDRS part 3 OFF medication scores at 96 weeks follow-up adjusting for baseline. The analysis will use a mixed-model approach incorporating information from all follow-up visits that adjusts for baseline Hoehn & Yahr status and the baseline raw value of each outcome measurement. Site will be included as a random effect to account for variability in outcomes between sites, and a random patient/subject effect will accommodate the correlation between repeated outcome measures on the same patient. A significance level of 5% will be used to judge significance for the primary outcome measure.

A planned secondary analysis will compare the difference in MDS-UPDRS part 3 OFF medication scores according to randomisation allocation at 96 weeks, with the scores at 48 weeks. An increase in the advantage at 96 weeks compared to 48 weeks would be evidence that the active drug was slowing down disease deterioration rather than having symptomatic effects only. This could translate to a major population advantage in terms of reduction of morbidity and mortality.

Analyses of the remaining secondary/exploratory outcomes will be undertaken similarly for the difference between groups according to treatment allocation at 48 and 96 weeks follow up adjusting

For peer review only

for baseline values of each outcome, and confounding factors such as LED differences between groups.

Further exploratory analyses will consider whether exenatide can be thought of as disease modifying by comparing slopes between groups at pre-specified periods.

A sensitivity (per protocol) analysis will be performed for the primary outcome measure and will only include those participants who completed the trial in accordance with the approved protocol.

Results on the primary efficacy outcome will be presented by stratum, according to Hoehn & Yahr stage (≤2.0 versus 2.5), and an interaction between Hoehn & Yahr and treatment will be added to the primary analysis model to investigate whether the effect of treatment differs according to the Hoehn & Yahr stage.

All analyses will be performed by the designated Trial Statistician.

Data Management

Data will be entered in the Exenatide-PD3 database by delegated staff at participating sites and members of the Exenatide-PD3 trial team at CCTU. Participants will be given a unique trial PIN (Exnnn). Data will be entered under the PIN onto the central database (InferMed's MACRO stored on the servers based at UCL). The database will be password protected and only accessible to members of the Exenatide-PD3 trial team and external regulators if requested. Video recordings of the MDS-UPDRS will be uploaded onto a secure cloud held by Machine Medicines Technologies (MMT) and used for quality control purposes. Appropriate contractual agreements covering data protection are in place with MMT. All data storage will adhere to GDPR and the Data Protection Act 2018.

An Independent Data Monitoring Committee (IDMC) will be convened including at least 3 individuals independent from the trial team and sponsor who have experience in the conduct of clinical trials for PD. The IDMC will review the trial results and make a recommendation to the Trial Steering Committee (TSC) regarding continuation/ stopping of the trial based on safety data. A statistician independent of the Exenatide-PD3 Trial Team at CCTU will generate summaries of accumulating trial data for the IDMC to review.

UCL is the trial sponsor and has delegated the duties as sponsor to CCTU via a signed letter of delegation. The trial sponsor will take on responsibility for securing the arrangements to initiate, manage and finance the trial. Trial oversight is intended to preserve the integrity of the trial by independently verifying processes and prompting corrective action where necessary. In multi-centre trials this oversight is considered and described both overall and for each recruiting centre by exploring the trial dataset or performing site visits. The Trial Team (TT) will assist with developing the design, co-ordination and day to day operational issues in the management of the trial, including budget management. The Trial Management Group (TMG) will assist with developing the design, co-ordination and strategic management of the trial. The Independent Trial Steering Committee (TSC) is the independent group responsible for oversight of the trial in order to safeguard the interests of trial participants. The TSC will provide advice to the Chief Investigator (CI), CCTU, the funder and sponsor on all aspects of the trial through its independent Chair. The Independent Data Monitoring Committee (IDMC) is the only oversight body that has access to unblinded accumulating comparative data. The IDMC will be responsible for safeguarding the interests of trial participants, monitoring the accumulating data and making recommendations to the TSC on whether the trial should continue as planned. The membership, frequency of meetings, activity (including trial conduct and data review) and authority of each committee will be covered in their respective terms of reference.

For peer review only

Ethics and Dissemination

The trial protocol, all informed consent forms and any material to be given to the prospective participant have received REC (initial date of approval 15/10/2019, REC reference no.19/SC/0447), and other regulatory approvals (EudraCT 2018-003028-35). Further, the trial was registered in clinicatrials.gov NCT004232969 and in ISRCTN (reference 14552789). Subsequent amendments to these documents will be submitted for further approval. The same/amended documents will be submitted for additional local permissions at each clinical site.

This is a Clinical Trial of an IMP as defined by the EU Directive 2001/20/EC. Therefore, a clinical trial authorisation (CTA) is required in the UK and the trial protocol will therefore be submitted to the UK regulatory authority (MHRA). The progress of the trial, safety issues and reports, including expedited reporting will be reported to the MHRA as required. The protocol, participant information sheet (PIS) and informed consent forms on local headed paper, the REC/HRA and MHRA approvals, schedules of funding and activity (and other trial documentation as needed) have been submitted to the relevant NHS Trust R&D department of each participating site or to other local departments for approval.

Participants will be provided with a PIS and given time to read it fully. Following a discussion with a medical qualified investigator or suitable trained and authorised delegate, any questions will be satisfactorily answered and if the participant is willing to participate, written informed consent will be obtained (Supplementary Materials 2). During the consent process it will be emphasised that the participant is free to refuse to participate in all or any aspect of the trial, at any time and for any reason, without affecting their treatment. The risk/benefit profile of the trial will be regularly monitored. Consent will be re-sought if new information becomes available that affects the participant’s consent in any way.

The rights of the participant to refuse to participate in the trial without giving a reason will be respected and after the participant has entered the trial, the clinician remains free to give alternative treatment to that specified in the protocol, at any stage. The participant remains free to change their mind at any time about the protocol treatment and follow-up without giving a reason and without prejudicing their further treatment. All participants will be made aware of the known adverse reactions.

Discussion

A parallel group design with a washout period has been used previously in the evaluation of potential neuroprotective agents [25,26]. and this was chosen as the design for the previous phase 2 trial. This design is subject to possible long duration symptomatic effects and a lengthy washout period potentially impacts on patient retention and cannot necessarily distinguish a true neuroprotective effect from a symptomatic effect (in view of preservation of healthy behaviours with long term impacts such as exercise) [27]. An alternative approach which we have adopted here is a “Long term simple” design, with longer term follow up to look for a cumulative advantage emerging with prolonged treatment exposure, given the natural history of PD being that of progressive accumulation of motor and non-motor disability [28]. This design helps build on the previous successful clinical trials of exenatide which have introduced a novel, cost effective way of evaluating the potential for disease modifying drugs in PD by recruiting patients already in receipt of conventional dopaminergic treatment, rather than restricting recruitment to incident cases yet to receive dopaminergic treatment. Using this approach, we have successfully demonstrated the

For peer review only

potential for rapid recruitment, and improved retention of participants enabling more complete follow up, and a statistically significant advantage in motor scores in people randomised to exenatide over a 48-week period of treatment exposure. We have considered that an exposure period of 96 weeks would allow exploration of long-term effects of exenatide exposure, while being the maximum period that participants would be willing to accept being allocated placebo. Furthermore, this will provide the opportunity to evaluate whether the 48-week data previously published can be replicated and whether effects at 96 weeks are similar to or greater than those seen at 48 weeks and other earlier timepoints.

Many trials have attempted to evaluate the potential for disease modification using drugs with broad mechanisms of action. The majority have either failed to demonstrate clinical efficacy or provided inconclusive results. Some pertinent reasons for this are a failure of the investigated agent to reach and engage its target and lack of objective measures of true clinical disease progression. Clinical endpoints such as the MDS-UPDRS scale are necessary to ultimately confirm relevance; however, these scales lack sensitivity for capturing disease modification unless very long term follow up data are collected. While our approach of following patients for 2 years in this trial will partially mitigate some of this, the addition and to an extent validation of more detailed approaches through a number of sub studies (imaging, CSF analysis for target engagement and drug levels, and device assisted measurements of real-life motor function) could ultimately provide more holistic and definitive metrics for determining if exenatide does in fact deliver disease modification in PD. This more comprehensive approach to approaching assessments and the consistent signal of benefit noted in two earlier trials provide grounds for optimism that the primary outcome will be achievable. The study opened to recruitment in January 2020 and we expect completion of study analysis by Q3 2024.

Author Contributions:

The study concept and design was conceived by TF. NV and CG will conduct screening and data collection. GA, MC, KM, KC, HM, SH, PL, DA, CC, MH, MS, GD, RC, CL, SDD, AY, LR, RG, JD, RH and VL assisted on drafting the protocol. Analysis will be performed by SS, and data entry by AK. NV prepared the first draft of the manuscript. RG served as a patient adviser for the design of the study protocol. All authors provided edits and revised the manuscript for intellectual content.

Acknowledgement

We are grateful to all patients contributing to the design of this study. We thank the Cure Parkinson’s Trust and Parkinson’s UK for their assistance in the setup and increasing the awareness of this study.

Funding Statement

This work was supported by NIHR grant number 16/167/19. The Cure Parkinson’s Trust funded the DATScan Imaging substudy grant number BY-TF021 & the Remote Monitoring substudy grant number MH011.

For peer review only

Competing Interest Statement

None declared

Data Statement

Not applicable at this stage.

Word Count 5729

For peer review only

References:

1 Ray Dorsey E, Elbaz A, Nichols E, *et al.* Global, regional, and national burden of Parkinson's disease, 1990–2016: a systematic analysis for the Global Burden of Disease Study 2016. *Lancet Neurol* 2018;**17**:939–53. doi:10.1016/S1474-4422(18)30295-3

2 Vijiaratnam N, Evans AH. Treatment advances in Parkinson disease: Same script different cast? *J Clin Neurosci* 2018;**47**:354–5. doi:10.1016/j.jocn.2017.10.009

3 Vijiaratnam N, Foltynie T. Therapeutic Strategies to Treat or Prevent Off Episodes in Adults with Parkinson's Disease. *Drugs* 2020;**80**:775–96. doi:10.1007/s40265-020-01310-2

4 Kalia L V., Lang AE. Parkinson's disease. Lancet. 2015;**386**:896–912. doi:10.1016/S0140-6736(14)61393-3

5 Htike ZZ, Zaccardi F, Papamargaritis D, *et al.* Efficacy and safety of glucagon-like peptide-1 receptor agonists in type 2 diabetes: A systematic review and mixed-treatment comparison analysis. *Diabetes, Obes Metab* 2017;**19**:524–36. doi:10.1111/dom.12849

6 Perry T, Lahiri DK, Chen D, *et al.* A Novel Neurotrophic Property of Glucagon-Like Peptide 1: A Promoter of Nerve Growth Factor-Mediated Differentiation in PC12 Cells. *J Pharmacol Exp Ther* 2002;**300**:958–66. doi:10.1124/jpet.300.3.958

7 Perry T, Haughey NJ, Mattson MP, *et al.* Protection and Reversal of Excitotoxic Neuronal Damage by Glucagon-Like Peptide-1 and Exendin-4. *J Pharmacol Exp Ther* 2002;**302**:881–8. doi:10.1124/jpet.102.037481

8 Aviles-Olmos I, Limousin P, Lees A, *et al.* Parkinson's disease, insulin resistance and novel agents of neuroprotection. *Brain* 2013;**136**:374–84. doi:10.1093/brain/aws009

9 Perry T, Lahiri DK, Sambamurti K, *et al.* Glucagon-like peptide-1 decreases endogenous amyloid-β peptide (Aβ) levels and protects hippocampal neurons from death induced by Aβ and iron. *J Neurosci Res* 2003;**72**:603–12. doi:10.1002/jnr.10611

10 Yamamoto H, Lee CE, Marcus JN, *et al.* Glucagon-like peptide-1 receptor stimulation increases blood pressure and heart rate and activates autonomic regulatory neurons. *J Clin Invest* 2002;**110**:43–52. doi:10.1172/JCI15595

11 Drucker DJ, Philippe J, Mojsov S, *et al.* Glucagon-like peptide I stimulates insulin gene expression and increases cyclic AMP levels in a rat islet cell line. *Proc Natl Acad Sci U S A* 1987;**84**:3434–8. doi:10.1073/pnas.84.10.3434

12 Brauer R, Wei L, Ma T, *et al.* Diabetes medications and risk of Parkinson's disease: a cohort study of patients with diabetes. *Brain* 2020;**143**:3067–76. doi:10.1093/brain/awaa262

13 Aviles-Olmos I, Dickson J, Kefalopoulou Z, *et al.* Exenatide and the treatment of patients with Parkinson's disease. *J Clin Invest* 2013;**123**:2730–6.

14 Athauda D, Maclagan K, Skene SS, *et al.* Exenatide once weekly versus placebo in Parkinson's disease: a randomised, double-blind, placebo-controlled trial. *Lancet* 2017;**390**:1664–75. doi:10.1016/S0140-6736(17)31585-4

15 Athauda D, Gulyani S, Karnati HK, *et al.* Utility of Neuronal-Derived Exosomes to Examine Molecular Mechanisms That Affect Motor Function in Patients with Parkinson Disease: A Secondary Analysis of the Exenatide-PD Trial. *JAMA Neurol* 2019;**76**:420–9. doi:10.1001/jamaneurol.2018.4304

16 Crocker JC, Ricci-Cabello I, Parker A, *et al.* Impact of patient and public involvement on

enrolment and retention in clinical trials: Systematic review and meta-analysis. *BMJ* 2018;**363**:k4738. doi:10.1136/bmj.k4738

17 https://www.nihr.ac.uk/documents/improving-inclusion-of-under-served-groups-in-clinical-research-guidance-from-include-project/25435.

18 GIBB WRG, LEES AJ. THE SIGNIFICANCE OF THE LEWY BODY IN THE DIAGNOSIS OF IDIOPATHIC PARKINSON'S DISEASE. *Neuropathol Appl Neurobiol* 1989;**15**:27–44. doi:10.1111/j.1365-2990.1989.tb01147.x

19 Athauda D, Maclagan K, Budnik N, *et al.* Post hoc analysis of the Exenatide-PD trial-Factors that predict response. *Eur J Neurosci* 2019;**49**:410–21. doi:10.1111/ejn.14096

20 Tomlinson CL, Stowe R, Patel S, *et al.* Systematic review of levodopa dose equivalency reporting in Parkinson's disease. *Mov Disord* 2010;**25**:2649–53. doi:10.1002/mds.23429

21 Del Din S, Galna B, Godfrey A, *et al.* Analysis of Free-Living Gait in Older Adults with and Without Parkinson's Disease and with and Without a History of Falls: Identifying Generic and Disease-Specific Characteristics. *Journals Gerontol - Ser A Biol Sci Med Sci* 2019;**74**:500–6. doi:10.1093/gerona/glx254

22 Del Din S, Godfrey A, Galna B, *et al.* Free-living gait characteristics in ageing and Parkinson's disease: impact of environment and ambulatory bout length. *J Neuroeng Rehabil* 2016;**13**:46. doi:10.1186/s12984-016-0154-5

23 Del Din S, Yarnall AJ, Barber TR, *et al.* Continuous Real-World Gait Monitoring in Idiopathic REM Sleep Behavior Disorder. *J Parkinsons Dis* 2020;**10**:283–99. doi:10.3233/JPD-191773

24 Shulman LM, Gruber-Baldini AL, Anderson KE, *et al.* The clinically important difference on the unified parkinson's disease rating scale. *Arch Neurol* 2010;**67**:64–70. doi:10.1001/archneurol.2009.295

25 Datatop: A multicenter controlled clinical trial in early parkinson's disease: Parkinson study group. *Arch Neurol* 1989;**46**:1052–60. doi:10.1001/archneur.1989.00520460028009

26 Olanow CW, Schapira AH, LeWitt PA, *et al.* TCH346 as a neuroprotective drug in Parkinson's disease: a double-blind, randomised, controlled trial. *Lancet Neurol* 2006;**5**:1013–20. doi:10.1016/S1474-4422(06)70602-0

27 Ward CD. Does selegiline delay progression of Parkinson's disease? A critical re-evaluation of the DATATOP study. *J Neurol Neurosurg Psychiatry* 1994;**57**:217–20. doi:10.1136/jnnp.57.2.217

28 Thibault L, Rascol O, Corvol JC, *et al.* New perspectives on study designs for evaluating neuroprotection in Parkinson's disease. *Mov Disord* 2017;**32**:1365–70. doi:10.1002/mds.27055

Figure legend

Figure 1: Outline of Trial design

For peer review only

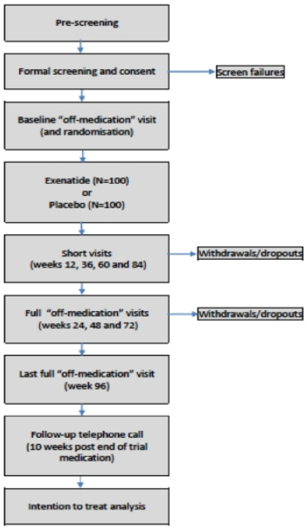

Figure 1: Outline of Trial design

209x296mm (400 x 400 DPI)

EUROPEAN COMMISSION
ENTERPRISE AND INDUSTRY DIRECTORATE-GENERAL

Consumer goods
Pharmaceuticals

Brussels, 03 February 2010
ENTR/F/2/AM/an D(2010) 3374

EudraLex

The Rules Governing Medicinal Products in the European Union

Volume 4

EU Guidelines to

Good Manufacturing Practice

Medicinal Products for Human and Veterinary Use
Annex 13

Investigational Medicinal Products

Document History	
Revision to reinforce the principle of independence between production and quality control functions in cases where the number of personnel involved is small.<br><br>Changes to sections 36 and 37 to supplement, for investigational medicinal products, the guidance for reference and retention samples given in Annex 19.<br><br>An additional note has been introduced to clarify the meaning of “reconstitution” as referred to in article 9.2 of Directive 2005/28/EC.<br><br>The content of the Batch Certificate referred to in Art. 13(3) of Directive 2001/20/EC, agreed following a separate public consultation, has been added as an attachment.<br><br>A few editorial changes have been made to sections not consulted upon in the interests of updating references and consistency with terminology used throughout the GMP Guide.	February 2008
Public consultation	April 2008 until January 2009
Adopted by the European Commission	31 January 2010
Deadline for coming into operation	31 July 2010

For peer review only - http://bmjopen.bmj.com/site/about/guidelines.xhtml

PRINCIPLE

Investigational medicinal products should be produced in accordance with the principles and the detailed guidelines of Good Manufacturing Practice for Medicinal Products (The Rules Governing Medicinal Products in The European Community, Volume IV). Other guidelines published by the European Commission should be taken into account where relevant and as appropriate to the stage of development of the product. Procedures need to be flexible to provide for changes as knowledge of the process increases, and appropriate to the stage of development of the product.

In clinical trials there may be added risk to participating subjects compared to patients treated with marketed products. The application of GMP to the manufacture of investigational medicinal products is intended to ensure that trial subjects are not placed at risk, and that the results of clinical trials are unaffected by inadequate safety, quality or efficacy arising from unsatisfactory manufacture. Equally, it is intended to ensure that there is consistency between batches of the same investigational medicinal product used in the same or different clinical trials, and that changes during the development of an investigational medicinal product are adequately documented and justified.

The production of investigational medicinal products involves added complexity in comparison to marketed products by virtue of the lack of fixed routines, variety of clinical trial designs, consequent packaging designs, and the need, often, for randomisation and blinding and increased risk of product cross-contamination and mix up. Furthermore, there may be incomplete knowledge of the potency and toxicity of the product and a lack of full process validation, or, marketed products may be used which have been re-packaged or modified in some way. These challenges require personnel with a thorough understanding of, and training in, the application of GMP to investigational medicinal products. Co-operation is required with trial sponsors who undertake the ultimate responsibility for all aspects of the clinical trial including the quality of investigational medicinal products. The increased complexity in manufacturing operations requires a highly effective quality system.

The Annex also includes guidance on ordering, shipping, and returning clinical supplies, which are at the interface with, and complementary to, guidelines on Good Clinical Practice.

Notes

Non-investigational medicinal product[1]

Products other than the test product, placebo or comparator may be supplied to subjects participating in a trial. Such products may be used as support or escape medication for preventative, diagnostic or therapeutic reasons and/or needed to ensure that adequate medical care is provided for the subject. They may also be used in accordance with the protocol to induce a physiological response. These products do not fall within the definition of investigational medicinal products and may be supplied by the sponsor, or the investigator. The sponsor should ensure that they are in accordance with the notification/request for authorisation to conduct the trial and that they are of appropriate quality for the purposes of the trial taking into account the source of the materials, whether or not they are the subject of

[1] Further information can be found in the European Commission's Guidance on Investigational Medicinal Products (IMPs) and other Medicinal Products used in Clinical Trials

For peer review only

a marketing authorisation and whether they have been repackaged. The advice and involvement of a Qualified Person is recommended in this task.

Manufacturing authorisation and reconstitution

Both the total and partial manufacture of investigational medicinal products, as well as the various processes of dividing up, packaging or presentation, is subject to the authorisation referred to in Article 13(1) Directive 2001/20/EC, cf. Article 9(1) Directive 2005/28/EC. This authorisation, however, shall not be required for reconstitution under the conditions set out in Article 9(2) Directive 2005/28/EC. For the purpose of this provision, reconstitution shall be understood as a simple process of:

- dissolving or dispersing the investigational medicinal product for administration of the product to a trial subject,
- or, diluting or mixing the investigational medicinal product(s) with some other substance(s) used as a vehicle for the purposes of administering it,

Reconstitution is not mixing several ingredients, including the active substance, together to produce the investigational medicinal product.

An investigational medicinal product must exist before a process can be defined as reconstitution.

The process of reconstitution has to be undertaken as soon as practicable before administration.

This process has to be defined in the clinical trial application / IMP dossier and clinical trial protocol, or related document, available at the site.

GLOSSARY

Blinding

A procedure in which one or more parties to the trial are kept unaware of the treatment assignment(s). Single-blinding usually refers to the subject(s) being unaware, and double-blinding usually refers to the subject(s), investigator(s), monitor, and, in some cases, data analyst(s) being unaware of the treatment assignment(s). In relation to an investigational medicinal product, blinding shall mean the deliberate disguising of the identity of the product in accordance with the instructions of the sponsor. Unblinding shall mean the disclosure of the identity of blinded products.

Clinical trial

Any investigation in human subjects intended to discover or verify the clinical, pharmacological and/or other pharmacodynamic effects of an investigational product(s) and/or to identify any adverse reactions to an investigational product(s), and/or to study absorption, distribution, metabolism, and excretion of one or more investigational medicinal product(s) with the object of ascertaining its/their safety and/or efficacy.

For peer review only

Comparator product

An investigational or marketed product (i.e. active control), or placebo, used as a reference in a clinical trial.

Investigational medicinal product

A pharmaceutical form of an active substance or placebo being tested or used as a reference in a clinical trial, including a product with a marketing authorisation when used or assembled (formulated or packaged) in a way different from the authorised form, or when used for an unauthorised indication, or when used to gain further information about the authorised form.

Investigator

A person responsible for the conduct of the clinical trial at a trial site. If a trial is conducted by a team of individuals at a trial site, the investigator is the responsible leader of the team and may be called the principal investigator.

Manufacturer/importer of Investigational Medicinal Products

Any person engaged in activities for which the authorisation referred to in Article 13(1) of Directive 2001/20/EC is required.

Order

Instruction to process, package and/or ship a certain number of units of investigational medicinal product(s).

Product Specification File

A reference file containing, or referring to files containing, all the information necessary to draft the detailed written instructions on processing, packaging, quality control testing, batch release and shipping of an investigational medicinal product.

Randomisation

The process of assigning trial subjects to treatment or control groups using an element of chance to determine the assignments in order to reduce bias.

Randomisation Code

A listing in which the treatment assigned to each subject from the randomisation process is identified.

Shipping

The operation of packaging for shipment and sending of ordered medicinal products for clinical trials.

Sponsor

An individual, company, institution or organisation which takes responsibility for the initiation, management and/or financing of a clinical trial.

For peer review only

QUALITY MANAGEMENT

1. The Quality System, designed, set up and verified by the manufacturer or importer, should be described in written procedures available to the sponsor, taking into account the GMP principles and guidelines applicable to investigational medicinal products.

2. The product specifications and manufacturing instructions may be changed during development but full control and traceability of the changes should be maintained.

PERSONNEL

3. All personnel involved with investigational medicinal products should be appropriately trained in the requirements specific to these types of product.

Even in cases where the number of staff involved is small, there should be, for each batch, separate people responsible for production and quality control.

4. The Qualified Person should ensure that there are systems in place that meet the requirements of GMP and should have a broad knowledge of pharmaceutical development and clinical trial processes. Guidance for the Qualified Person in connection with the certification of investigational medicinal products is given in paragraphs 38 to 41.

PREMISES AND EQUIPMENT

5. The toxicity, potency and sensitising potential may not be fully understood for investigational medicinal products and this reinforces the need to minimise all risks of cross-contamination. The design of equipment and premises, inspection / test methods and acceptance limits to be used after cleaning should reflect the nature of these risks. Consideration should be given to campaign working where appropriate. Account should be taken of the solubility of the product in decisions about the choice of cleaning solvent.

DOCUMENTATION

Specifications and instructions

6. Specifications (for starting materials, primary packaging materials, intermediate, bulk products and finished products), manufacturing formulae and processing and packaging instructions should be as comprehensive as possible given the current state of knowledge. They should be periodically re-assessed during development and updated as necessary. Each new version should take into account the latest data, current technology used, regulatory and pharmacopoeial requirements, and should allow traceability to the previous document. Any changes should be carried out according to a written procedure, which should address any implications for product quality such as stability and bio equivalence.

For peer review only

7. Rationales for changes should be recorded and the consequences of a change on product quality and on any on-going clinical trials should be investigated and documented[2].

Order

8. The order should request the processing and/or packaging of a certain number of units and/or their shipping and be given by or on behalf of the sponsor to the manufacturer. It should be in writing (though it may be transmitted by electronic means), and precise enough to avoid any ambiguity. It should be formally authorised and refer to the Product Specification File and the relevant clinical trial protocol as appropriate.

Product Specification File

9. The Product Specification File (see glossary) should be continually updated as development of the product proceeds, ensuring appropriate traceability to the previous versions. It should include, or refer to, the following documents:

- Specifications and analytical methods for starting materials, packaging materials;
- Intermediate, bulk and finished product;
- Manufacturing methods;
- In-process testing and methods;
- Approved label copy;
- Relevant clinical trial protocols and randomisation codes, as appropriate;
- Relevant technical agreements with contract givers, as appropriate;
- Stability data;
- Storage and shipment conditions.

The above listing is not intended to be exclusive or exhaustive. The contents will vary depending on the product and stage of development. The information should form the basis for assessment of the suitability for certification and release of a particular batch by the Qualified Person and should therefore be accessible to him/her. Where different manufacturing steps are carried out at different locations under the responsibility of different Qualified Persons, it is acceptable to maintain separate files limited to information of relevance to the activities at the respective locations.

Manufacturing Formulae and Processing Instructions

10. For every manufacturing operation or supply there should be clear and adequate written instructions and written records. Where an operation is not repetitive it may not be

---

[2] Guidance on changes that require the request of a substantial amendment to the IMP dossier submitted to the Competent Authorities is given in the CHMP guideline on the Requirements to the Chemical and Pharmaceutical Quality Documentation Concerning Investigational Medicinal Products in Clinical Trials

For peer review only - http://bmjopen.bmj.com/site/about/guidelines.xhtml

necessary to produce Master Formulae and Processing Instructions. Records are particularly important for the preparation of the final version of the documents to be used in routine manufacture once the marketing authorisation is granted.

11. The information in the Product Specification File should be used to produce the detailed written instructions on processing, packaging, quality control testing, storage conditions and shipping.

Packaging Instructions

12. Investigational medicinal products are normally packed in an individual way for each subject included in the clinical trial. The number of units to be packaged should be specified prior to the start of the packaging operations, including units necessary for carrying out quality control and any retention samples to be kept. Sufficient reconciliations should take place to ensure the correct quantity of each product required has been accounted for at each stage of processing.

Processing, testing and packaging batch records

13. Batch records should be kept in sufficient detail for the sequence of operations to be accurately determined. These records should contain any relevant remarks which justify the procedures used and any changes made, enhance knowledge of the product and develop the manufacturing operations.

14. Batch manufacturing records should be retained at least for the periods specified in Directive 2003/94/EC.

PRODUCTION

Packaging materials

15. Specifications and quality control checks should include measures to guard against unintentional unblinding due to changes in appearance between different batches of packaging materials.

Manufacturing operations

16. During development critical parameters should be identified and in-process controls primarily used to control the process. Provisional production parameters and in-process controls may be deduced from prior experience, including that gained from earlier development work. Careful consideration by key personnel is called for in order to formulate the necessary instructions and to adapt them continually to the experience gained in production. Parameters identified and controlled should be justifiable based on knowledge available at the time.

17. Production processes for investigational medicinal products are not expected to be validated to the extent necessary for routine production but premises and equipment are expected to be qualified. For sterile products, the validation of sterilising processes should be of the same standard as for products authorised for marketing. Likewise, when required, virus inactivation/removal and that of other impurities of biological origin should be demonstrated, to assure the safety of biotechnologically derived

For peer review only

products, by following the scientific principles and techniques defined in the available guidance in this area.

18. Validation of aseptic processes presents special problems when the batch size is small; in these cases the number of units filled may be the maximum number filled in production. If practicable, and otherwise consistent with simulating the process, a larger number of units should be filled with media to provide greater confidence in the results obtained. Filling and sealing is often a manual or semi-automated operation presenting great challenges to sterility so enhanced attention should be given to operator training, and validating the aseptic technique of individual operators.

Principles applicable to comparator product

19. If a product is modified, data should be available (e.g. stability, comparative dissolution, bioavailability) to demonstrate that these changes do not significantly alter the original quality characteristics of the product.

20. The expiry date stated for the comparator product in its original packaging might not be applicable to the product where it has been repackaged in a different container that may not offer equivalent protection, or be compatible with the product. A suitable use-by date, taking into account the nature of the product, the characteristics of the container and the storage conditions to which the article may be subjected, should be determined by or on behalf of the sponsor. Such a date should be justified and must not be later than the expiry date of the original package. There should be compatibility of expiry dating and clinical trial duration.

Blinding operations

21. Where products are blinded, systems should be in place to ensure that the blind is achieved and maintained while allowing for identification of "blinded" products when necessary, including the batch numbers of the products before the blinding operation. Rapid identification of product should also be possible in an emergency.

Randomisation code

22. Procedures should describe the generation, security, distribution, handling and retention of any randomisation code used for packaging investigational products, and code-break mechanisms. Appropriate records should be maintained.

Packaging

23. During packaging of investigational medicinal products, it may be necessary to handle different products on the same packaging line at the same time. The risk of product mix up must be minimised by using appropriate procedures and/or, specialised equipment as appropriate and relevant staff training.

24. Packaging and labelling of investigational medicinal products are likely to be more complex and more liable to errors (which are also harder to detect) than for marketed products, particularly when "blinded" products with similar appearance are used. Precautions against mis-labelling such as label reconciliation, line clearance, in process control checks by appropriately trained staff should accordingly be intensified.

For peer review only

25. The packaging must ensure that the investigational medicinal product remains in good condition during transport and storage at intermediate destinations. Any opening or tampering of the outer packaging during transport should be readily discernible.

Labelling

26. Table 1 summarises the contents of Articles 26-30 that follow. Labelling should comply with the requirements of Directive 2003/94/EC. The following information should be included on labels, unless its absence can be justified, e.g. use of a centralised electronic randomisation system:

(a) name, address and telephone number of the sponsor, contract research organisation or investigator (the main contact for information on the product, clinical trial and emergency unblinding);

(b) pharmaceutical dosage form, route of administration, quantity of dosage units, and in the case of open trials, the name/identifier and strength/potency;

(c) the batch and/or code number to identify the contents and packaging operation;

(d) a trial reference code allowing identification of the trial, site, investigator and sponsor if not given elsewhere;

(e) the trial subject identification number/treatment number and where relevant, the visit number;

(f) the name of the investigator (if not included in (a) or (d));

(g) directions for use (reference may be made to a leaflet or other explanatory document intended for the trial subject or person administering the product);

(h) "For clinical trial use only" or similar wording;

(i) the storage conditions;

(j) period of use (use-by date, expiry date or re-test date as applicable), in month/year format and in a manner that avoids any ambiguity.

(k) "keep out of reach of children" except when the product is for use in trials where the product is not taken home by subjects.

27. The address and telephone number of the main contact for information on the product, clinical trial and for emergency unblinding need not appear on the label where the subject has been given a leaflet or card which provides these details and has been instructed to keep this in their possession at all times.

28. Particulars should appear in the official language(s) of the country in which the investigational medicinal product is to be used. The particulars listed in Article 26 should appear on the primary packaging and on the secondary packaging (except for the cases described in Articles 29 and 30). The requirements with respect to the contents of the label on the primary and outer packaging are summarised in Table 1. Other languages may be included.

For peer review only

29. When the product is to be provided to the trial subject or the person administering the medication within a primary package together with secondary packaging that is intended to remain together, and the secondary packaging carries the particulars listed in Paragraph 26, the following information shall be included on the label of the primary package (or any sealed dosing device that contains the primary packaging):

    (a) name of sponsor, contract research organisation or investigator;

    (b) pharmaceutical dosage form, route of administration (may be excluded for oral solid dose forms), quantity of dosage units and in the case of open label trials, the name/identifier and strength/potency;

    (c) batch and/or code number to identify the contents and packaging operation;

    (d) a trial reference code allowing identification of the trial, site, investigator and sponsor if not given elsewhere;

    (e) the trial subject identification number/treatment number and where relevant, the visit number.

30. If the primary packaging takes the form of blister packs or small units such as ampoules on which the particulars required in Paragraph 26 cannot be displayed, secondary packaging should be provided bearing a label with those particulars. The primary packaging should nevertheless contain the following:

    (a) name of sponsor, contract research organisation or investigator;

    (b) route of administration (may be excluded for oral solid dose forms) and in the case of open label trials, the name/identifier and strength/potency;

    (c) batch and/or code number to identify the contents and packaging operation;

    (d) a trial reference code allowing identification of the trial, site, investigator and sponsor if not given elsewhere;

    (e) the trial subject identification number/treatment number and where relevant, the visit number;

31. Symbols or pictograms may be included to clarify certain information mentioned above. Additional information, warnings and/or handling instructions may be displayed.

32. For clinical trials with the characteristics identified in Article 14 of Directive 2001/20/EC, the following particulars should be added to the original container but should not obscure the original labelling:

    i) name of sponsor, contract research organisation or investigator;

    ii) trial reference code allowing identification of the trial site, investigator and trial subject.

33. If it becomes necessary to change the use-by date, an additional label should be affixed to the investigational medicinal product. This additional label should state the new use-by date and repeat the batch number. It may be superimposed on the old use-by date, but for quality control reasons, not on the original batch number. This operation should be

For peer review only

performed at an appropriately authorised manufacturing site. However, when justified, it may be performed at the investigational site by or under the supervision of the clinical trial site pharmacist, or other health care professional in accordance with national regulations. Where this is not possible, it may be performed by the clinical trial monitor(s) who should be appropriately trained. The operation should be performed in accordance with GMP principles, specific and standard operating procedures and under contract, if applicable, and should be checked by a second person. This additional labelling should be properly documented in both the trial documentation and in the batch records.

QUALITY CONTROL

34. As processes may not be standardised or fully validated, testing takes on more importance in ensuring that each batch meets its specification.

35. Quality control should be performed in accordance with the Product Specification File and in accordance with the information notified pursuant to Article 9(2) of Directive 2001/20/EC. Verification of the effectiveness of blinding should be performed and recorded.

36. Samples are retained to fulfill two purposes; firstly to provide a sample for analytical testing and secondly to provide a specimen of the finished product. Samples may therefore fall into two categories:

    Reference sample: a sample of a batch of starting material, packaging material, product contained in its primary packaging or finished product which is stored for the purpose of being analysed should the need arise. Where stability permits, reference samples from critical intermediate stages (e.g. those requiring analytical testing and release) or intermediates, which are transported outside of the manufacturer's control, should be kept.

    Retention sample: a sample of a packaged unit from a batch of finished product for each packaging run/trial period. It is stored for identification purposes. For example, presentation, packaging, labeling, leaflet, batch number, expiry date should the need arise.

    In many instances the reference and retention samples will be presented identically, i.e. as fully packaged units. In such circumstances, reference and retention samples may be regarded as interchangeable.Reference and retention samples of investigational medicinal product, including blinded product should be kept for at least two years after completion or formal discontinuation of the last clinical trial in which the batch was used, whichever period is the longer.

    Consideration should be given to keeping retention samples until the clinical report has been prepared to enable confirmation of product identity in the event of, and as part of an investigation into inconsistent trial results.

37. The storage location of Reference and Retention samples should be defined in a Technical Agreement between the sponsor and manufacturer(s) and should allow timely access by the competent authorities.

For peer review only

Reference samples of finished product should be stored within the EEA or in a third country where appropriate arrangements have been made by the Community with the exporting country to ensure that the manufacturer of the investigational medicinal product applies standards of good manufacturing practice at least equivalent to those laid down by the Community. In exceptional circumstances the reference samples of the finished product may be stored by the manufacturer in another third country, in which case this should be justified, and documented in a technical agreement between the sponsor, importer in the EEA and that third country manufacturer.

The reference sample should be of sufficient size to permit the carrying out, on, at least, two occasions, of the full analytical controls on the batch in accordance with the IMP dossier submitted for authorisation to conduct the clinical trial.

In the case of *retention samples*, it is acceptable to store information related to the final packaging as written or electronic records if such records provide sufficient information. In the case of the latter, the system should comply with the requirements of Annex 11.

RELEASE OF BATCHES

38. Release of investigational medicinal products (see paragraph 43) should not occur until after the Qualified Person has certified that the requirements of Article 13.3 of Directive 2001/20/EC have been met (see paragraph 39). The Qualified Person should take into account the elements listed in paragraph 40 as appropriate.

39. The duties of the Qualified Person in relation to investigational medicinal products are affected by the different circumstances that can arise and are referred to below. Table 2 summarises the elements that need to be considered for the most common circumstances:

(a) i) Product manufactured within EU but not subject to an EU marketing authorisation: the duties are laid down in article 13.3(a) of Directive 2001/20/EC.

(b) ii) Product sourced from the open market within EU in accordance with Article 80(b) of Directive 2001/83/EC and subject to an EU marketing authorisation, regardless of manufacturing origin: the duties are as described above, however, the scope of certification can be limited to assuring that the products are in accordance with the notification/request for authorisation to conduct the trial and any subsequent processing for the purpose of blinding, trial-specific packaging and labelling. The Product Specification File will be similarly restricted in scope (see 9).

(c) Product imported directly from a 3rd country: the duties are laid down in article 13.3(b) of Directive 2001/20/EC. Where investigational medicinal products are imported from a 3rd country and they are subject to arrangements concluded between the Community and that country, such as a Mutual Recognition Agreement (MRA), equivalent standards of Good Manufacturing Practice apply provided any such agreement is relevant to the product in question. In the absence of an MRA, the Qualified Person should determine that equivalent standards of Good Manufacturing Practice apply through knowledge of the quality system employed at the manufacturer. This knowledge is normally acquired through audit of the manufacturer's quality

systems. In either case, the Qualified Person may then certify on the basis of documentation supplied by the 3rd country manufacturer (see 40).

(d) For imported comparator products where adequate assurance cannot be obtained in order to certify that each batch has been manufactured to equivalent standards of Good Manufacturing Practice, the duty of the Qualified Person is defined in article 13.3(c) of Directive 2001/20/EC.

40. Assessment of each batch for certification prior to release may include as appropriate:

- batch records, including control reports, in-process test reports and release reports demonstrating compliance with the product specification file, the order, protocol and randomisation code. These records should include all deviations or planned changes, and any consequent additional checks or tests, and should be completed and endorsed by the staff authorised to do so according to the quality system;
- production conditions;
- the validation status of facilities, processes and methods;
- examination of finished packs;
- where relevant, the results of any analyses or tests performed after importation;
- stability reports;
- the source and verification of conditions of storage and shipment;
- audit reports concerning the quality system of the manufacturer;
- Documents certifying that the manufacturer is authorised to manufacture investigational medicinal products or comparators for export by the appropriate authorities in the country of export;
- where relevant, regulatory requirements for marketing authorisation, GMP standards applicable and any official verification of GMP compliance;
- all other factors of which the QP is aware that are relevant to the quality of the batch.

The relevance of the above elements is affected by the country of origin of the product, the manufacturer, and the marketed status of the product (with or without a marketing authorisation, in the EU or in a third country) and its phase of development. The sponsor should ensure that the elements taken into account by the qualified person when certifying the batch are consistent with the information notified pursuant to Article 9(2) of Directive 2001/20/EC. See also section 44.

41. Where investigational medicinal products are manufactured and packaged at different sites under the supervision of different Qualified Persons, the recommendations listed in Annex 16 to the GMP Guide should be followed as applicable.

42. Where, permitted in accordance with local regulations, packaging or labelling is carried out at the investigator site by, or under the supervision of a clinical trials pharmacist, or other health care professional as allowed in those regulations, the Qualified Person is not

For peer review only

required to certify the activity in question. The sponsor is nevertheless responsible for ensuring that the activity is adequately documented and carried out in accordance with the principles of GMP and should seek the advice of the Qualified Person in this regard.

SHIPPING

43. Investigational medicinal products should remain under the control of the sponsor until after completion of a two-step procedure: certification by the Qualified Person; and release by the sponsor for use in a clinical trial following fulfillment of the requirements of Article 9 (Commencement of a clinical trial) of Directive 2001/20/EC. Both steps should be recorded[3] and retained in the relevant trial files held by or on behalf of the sponsor. The Sponsor should ensure that the details set out in the clinical trial application and considered by the Qualified Person are consistent with what is finally accepted by the Competent Authorities. Suitable arrangements to meet this requirement should be established. In practical terms, this can best be achieved through a change control process for the Product Specification File and defined in a Technical Agreement between the QP and the Sponsor.

44. Shipping of investigational products should be conducted according to instructions given by or on behalf of the sponsor in the shipping order.

45. De-coding arrangements should be available to the appropriate responsible personnel before investigational medicinal products are shipped to the investigator site.

46. A detailed inventory of the shipments made by the manufacturer or importer should be maintained. It should particularly mention the addressees' identification.

47. Transfers of investigational medicinal products from one trial site to another should remain the exception. Such transfers should be covered by standard operating procedures. The product history while outside of the control of the manufacturer, through for example, trial monitoring reports and records of storage conditions at the original trial site should be reviewed as part of the assessment of the product's suitability for transfer and the advice of the Qualified person should be sought. The product should be returned to the manufacturer, or another authorised manufacturer, for re-labelling, if necessary, and certification by a Qualified Person. Records should be retained and full traceability ensured.

COMPLAINTS

48. The conclusions of any investigation carried out in relation to a complaint which could arise from the quality of the product should be discussed between the manufacturer or importer and the sponsor (if different). This should involve the Qualified Person and those responsible for the relevant clinical trial in order to assess any potential impact on the trial, product development and on subjects.

---

[3] A harmonised format for batch certification to facilitate movement between Member States is provided in attachment 3.

For peer review only - http://bmjopen.bmj.com/site/about/guidelines.xhtml

RECALLS AND RETURNS

Recalls

49. Procedures for retrieving investigational medicinal products and documenting this retrieval should be agreed by the sponsor, in collaboration with the manufacturer or importer where different. The investigator and monitor need to understand their obligations under the retrieval procedure.

50. The Sponsor should ensure that the supplier of any comparator or other medication to be used in a clinical trial has a system for communicating to the Sponsor the need to recall any product supplied.

Returns

51. Investigational medicinal products should be returned on agreed conditions defined by the sponsor, specified in approved written procedures.

52. Returned investigational medicinal products should be clearly identified and stored in an appropriately controlled, dedicated area. Inventory records of the returned medicinal products should be kept.

DESTRUCTION

53. The Sponsor is responsible for the destruction of unused and/or returned investigational medicinal products. Investigational medicinal products should therefore not be destroyed without prior written authorisation by the Sponsor.

54. The delivered, used and recovered quantities of product should be recorded, reconciled and verified by or on behalf of the sponsor for each trial site and each trial period. Destruction of unused investigational medicinal products should be carried out for a given trial site or a given trial period only after any discrepancies have been investigated and satisfactorily explained and the reconciliation has been accepted. Recording of destruction operations should be carried out in such a manner that all operations may be accounted for. The records should be kept by the Sponsor.

55. When destruction of investigational medicinal products takes place a dated certificate of, or receipt for destruction, should be provided to the sponsor. These documents should clearly identify, or allow traceability to, the batches and/or patient numbers involved and the actual quantities destroyed.

For peer review only

TABLE 1: SUMMARY OF LABELLING DETAILS (§26 TO 30)

a) name, address and telephone number of the sponsor, contract research organisation or investigator (the main contact for information on the product, clinical trial and emergency unblinding);

(b) pharmaceutical dosage form, route of administration, quantity of dosage units, and in the case of open trials, the name/identifier and strength/potency;

(c) the batch and/or code number to identify the contents and packaging operation;

(d) a trial reference code allowing identification of the trial, site, investigator and sponsor if not given elsewhere;

(e) the trial subject identification number/treatment number and where relevant, the visit number;

(f) the name of the investigator (if not included in (a) or (d);

(g) directions for use (reference may be made to a leaflet or other explanatory document intended for the trial subject or person administering the product

(h) "for clinical trial use only" or similar wording;

(i) the storage conditions;

(j) period of use (use-by date, expiry date or re-test date as applicable), in month/year format and in a manner that avoids any ambiguity.

(k) "keep out of reach of children" except when the product is for use in trials where the product is not taken home by subjects.

GENERAL CASE
For both the primary and secondary packaging (§26)

Particulars a[4] to k

PRIMARY PACKAGE
Where primary and secondary packaging remain together throughout (§29)[5]

a[6] b[7] c d e

PRIMARY PACKAGE
Blisters or small packaging units (§30)[5]

a[6] b[7,8] c d e

For peer review only

---

4 The address and telephone number of the main contact for information on the product, clinical trial and for emergency unblinding need not appear on the label where the subject has been given a leaflet or card which provides these details and has been instructed to keep this in their possession at all times (§ 27).

5 When the outer packaging carries the particulars listed in Article 26.

6 The address and telephone number of the main contact for information on the product, clinical trial and for emergency unblinding need not be included.

7 Route of administration may be excluded for oral solid dose forms.

8 The pharmaceutical dosage form and quantity of dosage units may be omitted.

For peer review only - http://bmjopen.bmj.com/site/about/guidelines.xhtml

Table 2: BATCH RELEASE OF PRODUCTS

ELEMENTS TO BE TAKEN INTO ACCOUNT(3)	PRODUCT AVAILABLE IN THE EU		PRODUCT IMPORTED FROM THIRD COUNTRIES		
	**Product manufactured in EU without MA**	**Product with MA and available on EU market**	**Product without any EU MA**	**Product with a EU MA**	**Comparator where documentation certifying that each batch has been manufactured in conditions at least equivalent to those laid down in Directive 2003/94/EC cannot be obtained**
**BEFORE CLINICAL TRIAL PROCESSING**					
a) Shipping and storage conditions	Yes				
b) All relevant factors (1) showing that each batch has been manufactured and released in accordance with:<br>Directive 2003/94/EC, or<br>GMP standards at least equivalent to those laid down in Directive 2003/94/EC.	Yes<br>-		Yes (2)		
c) Documentation showing that each batch has been released within the EU according to EU GMP requirements (see Directive 2001/83/EC, article 51), or documentation showing that the product is available on the EU market and has been procured in accordance with article 80(b) of Directive 2001/83/EC.		Yes			
d) Documentation showing that the product is available on the local market and documentation to establish confidence in the local regulatory requirements for marketing authorisation and release for local use.					Yes
e) Results of all analysis, tests and checks performed to assess the quality of the imported batch according to:<br>the requirements of the MA (see Directive 2001/83/EC, article 51b), or<br>the Product Specification File, the Order, article 9.2 submission to the regulatory authorities.			-<br>Yes	Yes<br>-	-<br>Yes
Where these analyses and tests are not performed in the EU, this should be justified and the QP must certify that they have been carried out in accordance with GMP standards at least equivalent to those laid down in Directive 2003/94/EC.			Yes	Yes	Yes
**AFTER CLINICAL TRIAL PROCESSING**					
f) In addition to the assessment before clinical trial processing, all further relevant factors (1) showing that each batch has been processed for the purposes of blinding, trial-specific packaging, labelling and testing in accordance with:<br>Directive 2003/94/EC, or<br>GMP standards at least equivalent to those laid down in Directive 2003/94/EC.	Yes<br>-		Yes (2)		

For peer review only

For peer review only - http://bmjopen.bmj.com/site/about/guidelines.xhtml

ATTACHMENT 3

[LETTERHEAD OF MANUFACTURER]

Content of the Batch Certificate

Referred to in Art. 13.3 Directive 2001/20/EC

(1) Name(s) of product(s)/product identifier(s) as referred to in the clinical trial application, where applicable.

(2) EudraCT No(s) and sponsor protocol code number, when available.

(3) Strength

Identity (name) and amount per unit dose for all active substance(s) for each IMP (including placebo). The manner in which this information is provided should not unblind the study.

(4) Dosage form (pharmaceutical form)

(5) Package size (contents of container) and type (e.g. vials, bottles, blisters).

(6) Lot/batch number

(7) Expiry/retest/use by date

(8) Name and address of manufacturer where the Qualified Person issuing the certificate is located.

(9) Manufacturing Authorisation number for the site listed under item 8.

(10) Comments/remarks

(11) Any additional information considered relevant by the QP.

(12) Certification statement.

(13) "I hereby certify that this batch complies with the requirements of Article 13.3 of Directive 2001/20/EC "

(14) Name of the QP signing the certificate

(15) Signature

(16) Date of signature

Explanatory Note

Investigational medicinal products may not be used in a clinical trial in a member state of the European Economic Area until the completion of the two-step procedure referred to in section 43 of this Annex. The first step is the certification of each batch by the Qualified Person of the manufacturer or importer that the provisions of Article 13.3(a),

For peer review only

(b) or (c) of Directive 2001/20/EC have been complied with and documented in accordance with Art. 13.4 of the same Directive. According to Directive 2001/20/EC a batch of investigational medicinal product shall not have to undergo further checks in relation to the provisions of article 13.3(a), (b) or (c) of the same directive when it moves between Member States accompanied by batch certification signed by the Qualified Person. In order to facilitate the free movement of investigational medicinal products between Member States the content of these certificates should be in accordance with the above harmonised format. This format may also be used to certify batches destined for use within the Member State of the manufacturer or importer.

For peer review only

For peer review only - http://bmjopen.bmj.com/site/about/guidelines.xhtml

UCL COMPREHENSIVE CLINICAL TRIALS UNIT

NHS University College London Hospitals NHS Foundation Trust

Exenatide-PD3 Site Number: ___ ___ **Exenatide-PD3 Patient Identifiers:**
Screening ID: __ __ - __ __ __ __ __
Trial ID (Exnnn): __ __ __ __ __

Sponsor R&D Number: 18/0320 **Chief Investigator: Professor Tom Foltynie**

Exenatide-PD3 Informed Consent Form

A randomised, double blind, parallel group, placebo controlled, Phase 3 trial of Exenatide once weekly over 2 years as a potential disease modifying treatment for Parkinson's disease.

This Informed Consent Form is intended for consenting patients to take part in the Exenatide-PD3 trial.

	CONSENT FORM	Please initial
1	I have read the Exenatide-PD3 information sheet version (X.X)________dated (DD/MMM/YYYY) _______________ for this trial. I have had the opportunity to consider the information and ask questions that have been answered satisfactorily.	
2	I understand that my participation in the Exenatide-PD3 is voluntary and that I am free to withdraw at any time, without giving any reason and without my medical care or legal rights being affected.	
3	I understand that I may not benefit directly by participating in this trial. However, the research may help others in the future.	
4	I understand that relevant sections of my medical notes and data collected during the trial may be looked at by individuals from the sponsors' office (University College London), regulatory authorities, or from the NHS Trust or drug manufacturer, where it is relevant to my taking part in this research. I understand that these individuals have a duty of confidentiality towards me.	
5	I understand that one of my assessments at five of my visits will be video recorded, stored securely on a GDPR compliant server and securely transferred to Machine Medicines Technologies (MMT) for analysis. I understand that they will be used for quality control purposes, and to help improve PD assessments.	
6	I understand that I am required to attend five of my visits off of my normal PD medication, as stated in the Patient Information Sheet.	

For peer review only - http://bmjopen.bmj.com/site/about/guidelines.xhtml

UCL COMPREHENSIVE CLINICAL TRIALS UNIT

NHS University College London Hospitals NHS Foundation Trust

7	If the trial research team are unable to contact me at any time during the trial, I agree to the relevant sections of my medical records on NHS Digital (Spine) being accessed to obtain my contact details.	
8	I agree to my General Practitioner (GP) being informed of my participation in the Exenatide-PD3 trial.	
9	I agree that my data gathered in this trial will be stored in a secure facility (with limited access by individual from the sponsor's office (University College London) for 10 years after the completion of the trial as set out by the UK Medicines & Healthcare products Regulatory Agency.	
10	I give consent for my data and blood samples collected as part of the trial to be shared with other researchers and used in other ethically approved research following legal requirements to conceal my identity.	
11	I agree to take part in the Exenatide-PD3 Trial.	
12	I understand that one of my assessments at five of my visits will be recorded using an Electromagnetic Sensor (placed on my hand) and the information collected will be stored securely on a GDPR compliant server and securely transferred to The University Of York for analysis. *(Participating sites only).*	

Participant Name<br>________________________	Date (DD-MMM-YYYY)<br>_ _/ _ _ _/ _ _ _ _	Signature<br>______________________________
Person taking Consent<br>________________________	Date (DD-MMM-YYYY)<br>_ _/ _ _ _/ _ _ _ _	Signature<br>______________________________
Witness (if applicable)<br>________________________	Date (DD-MMM-YYYY)<br>_ _/ _ _ _/ _ _ _ _	Signature<br>______________________________

Once completed, please give 1 copy to the participant, keep 1 copy in the participant's medical records and file the original in the investigator site file.

For peer review only - http://bmjopen.bmj.com/site/about/guidelines.xhtml

Reporting checklist for protocol of a clinical trial.

Based on the SPIRIT guidelines.

Instructions to authors

Complete this checklist by entering the page numbers from your manuscript where readers will find each of the items listed below.

Your article may not currently address all the items on the checklist. Please modify your text to include the missing information. If you are certain that an item does not apply, please write "n/a" and provide a short explanation.

Upload your completed checklist as an extra file when you submit to a journal.

In your methods section, say that you used the SPIRITreporting guidelines, and cite them as:

Chan A-W, Tetzlaff JM, Gøtzsche PC, Altman DG, Mann H, Berlin J, Dickersin K, Hróbjartsson A, Schulz KF, Parulekar WR, Krleža-Jerić K, Laupacis A, Moher D. SPIRIT 2013 Explanation and Elaboration: Guidance for protocols of clinical trials. BMJ. 2013;346:e7586

		Reporting Item	Page Number
**Administrative information**			
Title	#1	Descriptive title identifying the study design, population, interventions, and, if applicable, trial acronym	1
Trial registration	#2a	Trial identifier and registry name. If not yet registered, name of intended registry	2
Trial registration: data set	#2b	All items from the World Health Organization Trial Registration Data Set	2
Protocol version	#3	Date and version identifier	16
Funding	#4	Sources and types of financial, material, and other support	16
Roles and responsibilities: contributorship	#5a	Names, affiliations, and roles of protocol contributors	16

For peer review only

Roles and responsibilities: sponsor contact information	#5b	Name and contact information for the trial sponsor	1
Roles and responsibilities: sponsor and funder	#5c	Role of study sponsor and funders, if any, in study design; collection, management, analysis, and interpretation of data; writing of the report; and the decision to submit the report for publication, including whether they will have ultimate authority over any of these activities	6
Roles and responsibilities: committees	#5d	Composition, roles, and responsibilities of the coordinating centre, steering committee, endpoint adjudication committee, data management team, and other individuals or groups overseeing the trial, if applicable (see Item 21a for data monitoring committee)	6
**Introduction**			
Background and rationale	#6a	Description of research question and justification for undertaking the trial, including summary of relevant studies (published and unpublished) examining benefits and harms for each intervention	3-4
Background and rationale: choice of comparators	#6b	Explanation for choice of comparators	3-4
Objectives	#7	Specific objectives or hypotheses	4
Trial design	#8	Description of trial design including type of trial (eg, parallel group, crossover, factorial, single group), allocation ratio, and framework (eg, superiority, equivalence, non-inferiority, exploratory)	6-7
**Methods: Participants, interventions, and outcomes**			
Study setting	#9	Description of study settings (eg, community clinic, academic hospital) and list of countries where data will be collected. Reference to where list of study sites can be obtained	6-7
Eligibility criteria	#10	Inclusion and exclusion criteria for participants. If applicable, eligibility criteria for study centres and individuals who will	8

For peer review only

		perform the interventions (eg, surgeons, psychotherapists)	
Interventions: description	#11a	Interventions for each group with sufficient detail to allow replication, including how and when they will be administered	12
Interventions: modifications	#11b	Criteria for discontinuing or modifying allocated interventions for a given trial participant (eg, drug dose change in response to harms, participant request, or improving / worsening disease)	12
Interventions: adherance	#11c	Strategies to improve adherence to intervention protocols, and any procedures for monitoring adherence (eg, drug tablet return; laboratory tests)	12
Interventions: concomitant care	#11d	Relevant concomitant care and interventions that are permitted or prohibited during the trial	12
Outcomes	#12	Primary, secondary, and other outcomes, including the specific measurement variable (eg, systolic blood pressure), analysis metric (eg, change from baseline, final value, time to event), method of aggregation (eg, median, proportion), and time point for each outcome. Explanation of the clinical relevance of chosen efficacy and harm outcomes is strongly recommended	9-10
Participant timeline	#13	Time schedule of enrolment, interventions (including any run-ins and washouts), assessments, and visits for participants. A schematic diagram is highly recommended (see Figure)	6-7
Sample size	#14	Estimated number of participants needed to achieve study objectives and how it was determined, including clinical and statistical assumptions supporting any sample size calculations	12
Recruitment	#15	Strategies for achieving adequate participant enrolment to reach target sample size	6
**Methods: Assignment of interventions (for controlled trials)**			
Allocation: sequence generation	#16a	Method of generating the allocation sequence (eg, computer-generated random numbers), and list of any factors for stratification. To reduce predictability of a random sequence, details of any planned restriction (eg, blocking) should be provided in a separate document that is unavailable to those who enrol participants or assign interventions	14

For peer review only

Allocation concealment mechanism	#16b	Mechanism of implementing the allocation sequence (eg, central telephone; sequentially numbered, opaque, sealed envelopes), describing any steps to conceal the sequence until interventions are assigned	14
Allocation: implementation	#16c	Who will generate the allocation sequence, who will enrol participants, and who will assign participants to interventions	14
Blinding (masking)	#17a	Who will be blinded after assignment to interventions (eg, trial participants, care providers, outcome assessors, data analysts), and how	14
Blinding (masking): emergency unblinding	#17b	If blinded, circumstances under which unblinding is permissible, and procedure for revealing a participant's allocated intervention during the trial	14
**Methods: Data collection, management, and analysis**			
Data collection plan	#18a	Plans for assessment and collection of outcome, baseline, and other trial data, including any related processes to promote data quality (eg, duplicate measurements, training of assessors) and a description of study instruments (eg, questionnaires, laboratory tests) along with their reliability and validity, if known. Reference to where data collection forms can be found, if not in the protocol	11
Data collection plan: retention	#18b	Plans to promote participant retention and complete follow-up, including list of any outcome data to be collected for participants who discontinue or deviate from intervention protocols	11
Data management	#19	Plans for data entry, coding, security, and storage, including any related processes to promote data quality (eg, double data entry; range checks for data values). Reference to where details of data management procedures can be found, if not in the protocol	11
Statistics: outcomes	#20a	Statistical methods for analysing primary and secondary outcomes. Reference to where other details of the statistical analysis plan can be found, if not in the protocol	13-14
Statistics: additional analyses	#20b	Methods for any additional analyses (eg, subgroup and adjusted analyses)	13-14

For peer review only

Statistics: analysis population and missing data	#20c	Definition of analysis population relating to protocol non-adherence (eg, as randomised analysis), and any statistical methods to handle missing data (eg, multiple imputation)	13-14
**Methods: Monitoring**			
Data monitoring: formal committee	#21a	Composition of data monitoring committee (DMC); summary of its role and reporting structure; statement of whether it is independent from the sponsor and competing interests; and reference to where further details about its charter can be found, if not in the protocol. Alternatively, an explanation of why a DMC is not needed	7
Data monitoring: interim analysis	#21b	Description of any interim analyses and stopping guidelines, including who will have access to these interim results and make the final decision to terminate the trial	7
Harms	#22	Plans for collecting, assessing, reporting, and managing solicited and spontaneously reported adverse events and other unintended effects of trial interventions or trial conduct	7
Auditing	#23	Frequency and procedures for auditing trial conduct, if any, and whether the process will be independent from investigators and the sponsor	7
**Ethics and dissemination**			
Research ethics approval	#24	Plans for seeking research ethics committee / institutional review board (REC / IRB) approval	14
Protocol amendments	#25	Plans for communicating important protocol modifications (eg, changes to eligibility criteria, outcomes, analyses) to relevant parties (eg, investigators, REC / IRBs, trial participants, trial registries, journals, regulators)	14
Consent or assent	#26a	Who will obtain informed consent or assent from potential trial participants or authorised surrogates, and how (see Item 32)	11-13
Consent or assent: ancillary studies	#26b	Additional consent provisions for collection and use of participant data and biological specimens in ancillary studies, if applicable	11-13
Confidentiality	#27	How personal information about potential and enrolled participants will be collected, shared, and maintained in order to protect confidentiality before, during, and after the trial	11-13

For peer review only

Declaration of interests	#28	Financial and other competing interests for principal investigators for the overall trial and each study site	16
Data access	#29	Statement of who will have access to the final trial dataset, and disclosure of contractual agreements that limit such access for investigators	15
Ancillary and post trial care	#30	Provisions, if any, for ancillary and post-trial care, and for compensation to those who suffer harm from trial participation	15
Dissemination policy: trial results	#31a	Plans for investigators and sponsor to communicate trial results to participants, healthcare professionals, the public, and other relevant groups (eg, via publication, reporting in results databases, or other data sharing arrangements), including any publication restrictions	15
Dissemination policy: authorship	#31b	Authorship eligibility guidelines and any intended use of professional writers	15
Dissemination policy: reproducible research	#31c	Plans, if any, for granting public access to the full protocol, participant-level dataset, and statistical code	15
**Appendices**			
Informed consent materials	#32	Model consent form and other related documentation given to participants and authorised surrogates	11
Biological specimens	#33	Plans for collection, laboratory evaluation, and storage of biological specimens for genetic or molecular analysis in the current trial and for future use in ancillary studies, if applicable	11

The SPIRIT checklist is distributed under the terms of the Creative Commons Attribution License CC-BY-ND 3.0. This checklist was completed on 14. December 2020 using https://www.goodreports.org/, a tool made by the EQUATOR Network in collaboration with Penelope.ai

For peer review only
